# Neutrophil efferocytosis in chronic inflammatory gastrointestinal diseases: mechanistic insights and therapeutic potential

**DOI:** 10.3389/fimmu.2026.1835168

**Published:** 2026-05-07

**Authors:** Si-ying Ye, Yi Qu, Jia-hao Zhang, Xi-han Wang, Jian-bo Wang, Ya-nan Xue, Ji-dong Liu

**Affiliations:** 1 Liaoning University of Traditional Chinese Medicine, Shenyang, China; 2 Liaoning University of Traditional Chinese Medicine Affiliated Second Hospital, Shenyang, China

**Keywords:** CD47-SIRPa signaling axis, efferocytosis, inflammation resolution, inflammatory bowel disease (IBD), macrophage polarization, MerTK/TAM receptors, neutrophils, nonalcoholic steatohepatitis (NASH)

## Abstract

Efferocytosis—the phagocytic clearance of apoptotic cells—is central to tissue homeostasis and the active resolution of inflammation. Although its mechanistic basis and disease relevance have been studied in isolation, no review has comprehensively integrated neutrophil efferocytosis mechanisms, their pathophysiological roles across major chronic inflammatory gastrointestinal diseases, and natural product-based therapeutic strategies—a gap this work addresses. We systematically describe the efferocytic recognition cascade, encompassing find-me signals, eat-me signals (including phosphatidylserine and the underappreciated plasminogen/M6P-IGF2R axis), and don’t-eat-me checkpoints (CD47-SIRPα). We clarify that efferocytosis is not restricted to M2-polarized macrophages—M0 and M1 macrophages also participate—but that polarization state critically determines the pro-resolving coupling of downstream signaling. We analyze shared and disease-specific efferocytic defect mechanisms in inflammatory bowel disease, chronic gastritis, NAFLD/NASH, and pancreatitis, identifying IL-10R signaling failure, receptor shedding, CD47 upregulation, and SPM deficiency as convergent pathological nodes. Against this backdrop, we critically evaluate natural product strategies—flavonoids, polyphenols, alkaloids, terpenoids, polysaccharides, and omega-3 fatty acids—targeting these nodes, with explicit grading of evidence levels. Translational challenges and the potential of single-cell sequencing, spatial transcriptomics, and patient-derived organoid co-culture systems are also discussed. Restoring efferocytosis represents a mechanistically grounded therapeutic frontier for chronic gastrointestinal disease.

## 1 Introduction

Neutrophils are core effector cells of the innate immune system, playing a key regulatory role in vascular inflammation (1). In the gastrointestinal tract, neutrophils participate in inflammatory responses through multiple mechanisms including phagocytosis, degranulation, reactive oxygen species (ROS) release, and neutrophil extracellular trap (NET) formation. However, neutrophils exhibit a dual role in gastrointestinal inflammation—serving as both critical participants in host defense and important mediators of tissue damage.

Inflammatory bowel disease (IBD), encompassing Crohn’s disease and ulcerative colitis, is characterized by aberrant immune responses leading to chronic inflammation (2). In ulcerative colitis (UC), neutrophil infiltration represents a core histological feature of disease activity. Neutrophil-related biomarkers, including calprotectin and lactoferrin, hold significant value in monitoring disease activity (3). Clinical studies confirm that complete resolution of mucosal neutrophils correlates with improved long-term clinical outcomes and serves as an independent predictor of the need for systemic corticosteroid therapy, hospitalization, and colectomy within 3 years in UC patients (4). The dichotomous functionality of neutrophils—participating in both microbial clearance and tissue damage—makes them key cells in IBD pathophysiology (5).

Efferocytosis, derived from the Latin “effere” (to bury), refers to the process by which professional phagocytes recognize, engulf, and degrade apoptotic cells (6). As a central mechanism for maintaining tissue homeostasis, efferocytosis not only clears billions of apoptotic cells generated daily but also exerts important immunoregulatory functions by inducing immune tolerance and promoting inflammation resolution. The process involves precisely coordinated molecular events: apoptotic cells expose “eat-me” signals such as phosphatidylserine (PS), which are recognized by phagocyte receptors (including MerTK, TIM-4, and integrins). Downregulation of “don’t-eat-me” signals (such as the CD47-SIRPα axis) permits phagocytosis, followed by phagosome-lysosome fusion to complete apoptotic cell degradation (6, 7).

When performed by M2-polarized or M0 macrophages, efferocytosis activates a pro-resolving transcriptional program characterized by IL-10 and TGF-β secretion and enhanced SPM biosynthesis, collectively promoting the active resolution of inflammation. Notably, this pro-resolving downstream coupling is not automatic: M1-polarized macrophages—which predominate in actively inflamed gastrointestinal tissues—can also perform efferocytosis but with attenuated activation of the IL-10/TGF-β/SPM axis, and may continue to secrete pro-inflammatory cytokines despite having engulfed apoptotic cells (6, 8). Therefore, the therapeutic goal in chronic inflammatory diseases is twofold: to restore efferocytic frequency and to restore the pro-resolving functional program that normally accompanies efferocytosis in tissue-homeostatic macrophages.

Neutrophils represent the most rapidly turned over leukocyte population in the body, and their efficient clearance following apoptosis is critical for inflammation resolution (9). Efferocytosis defects are a common pathological feature of multiple chronic inflammatory gastrointestinal diseases. In IBD, interleukin-10 receptor (IL-10R) signaling in innate immune cells is crucial for regulating anti-inflammatory macrophage function. Mice with IL-10R signaling defects develop severe colitis with profound impairments in anti-inflammatory macrophage generation and efferocytic capacity (10). The CD47-SIRPα signaling axis serves as a “don’t-eat-me” checkpoint of efferocytosis. Research has demonstrated that CD47 upregulation is associated with efferocytosis defects, and CD47-blocking antibody treatment can restore phagocytosis and reduce disease progression (11). Although initially studied in atherosclerosis, this mechanism may play similar roles in gastrointestinal inflammatory diseases.

Beyond IBD, efferocytosis dysfunction contributes to pathophysiology in other gastrointestinal disorders. In non-alcoholic steatohepatitis (NASH), neutrophil-driven inflammation involves complex immunometabolic networks (12). In pancreatitis, inflammasome activation and inflammatory response regulation are closely associated with disease progression (13). The role of macrophage efferocytosis in these conditions requires further investigation.

Recent advances have highlighted inflammation resolution as an actively regulated process rather than passive decay. SPMs are endogenous lipid mediators produced from ω-3 polyunsaturated fatty acid metabolism that actively terminate inflammatory responses through promoting neutrophil apoptosis, enhancing macrophage efferocytosis, and inhibiting pro-inflammatory cytokine production (8). Based on efferocytosis’s key role in inflammation resolution, therapeutic strategies targeting this process have emerged, including enhancing efferocytosis receptor function, blocking “don’t-eat-me” signals, promoting macrophage polarization, and supplementing SPMs (14). However, clinical translation faces challenges including target specificity, administration routes, and dose optimization.

This review aims to systematically elucidate the molecular mechanisms of neutrophil efferocytosis and its pathophysiological significance in chronic inflammatory gastrointestinal diseases, analyze the roles of efferocytosis defects in IBD, NAFLD/NASH, and pancreatitis, and explore therapeutic strategies targeting efferocytosis. Through comprehensive analysis of existing evidence, this review provides theoretical references for developing novel therapeutic strategies based on efferocytosis modulation and inflammation resolution.

## 2 Neutrophil efferocytosis: molecular mechanisms and immunological significance

### 2.1 Molecular mechanisms of efferocytosis

#### 2.1.1 Release of “Find-Me” signals

**Figure 1:**
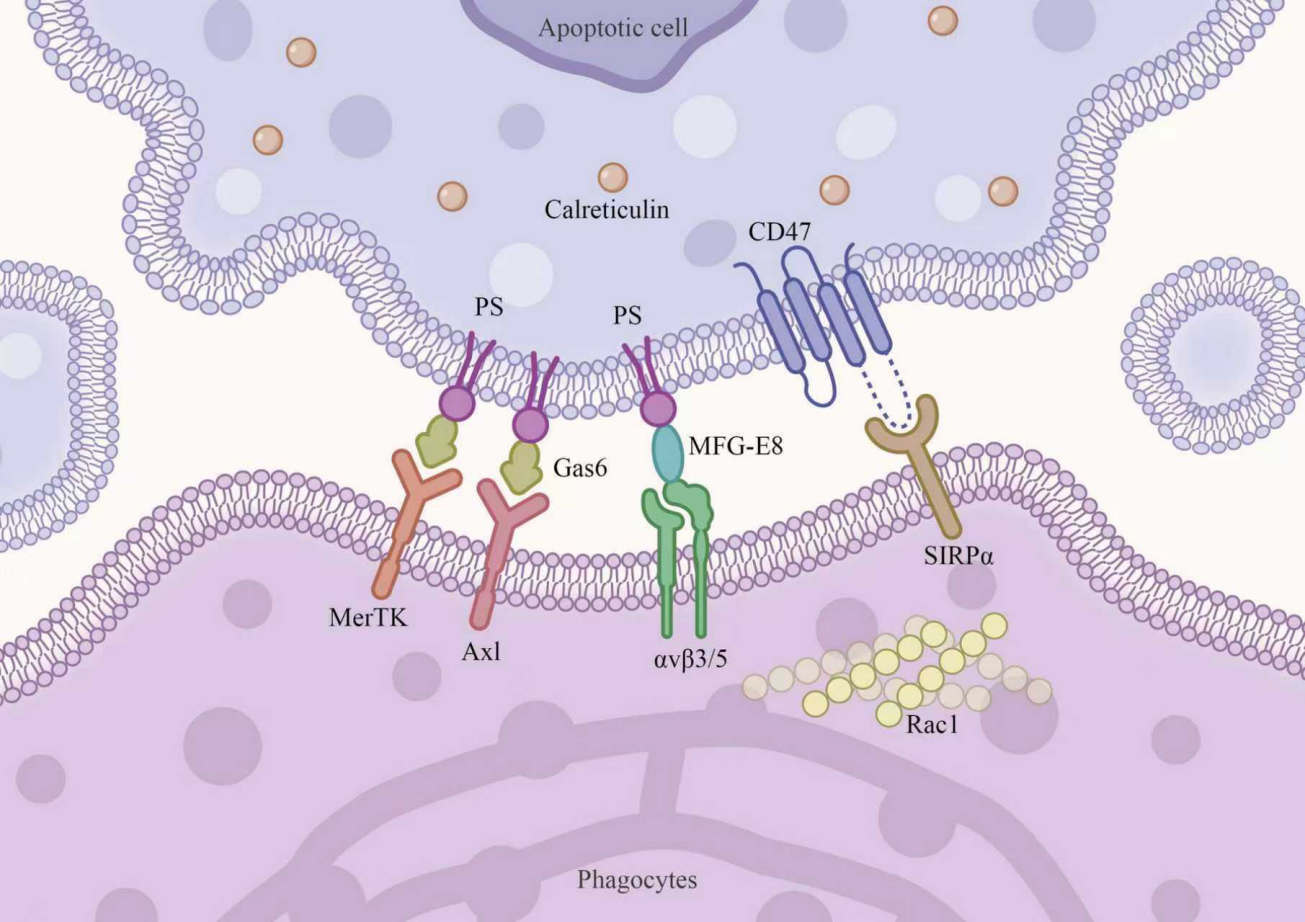
Molecular architecture of the efferocytic synapse between an apoptotic cell and a phagocyte.The apoptotic cell (upper) exposes phosphatidylserine (PS) on its outer membrane leaflet as a primary “eat-me” signal, which is recognized by phagocyte receptors either directly or via bridging proteins: MerTK and Axl engage PS through the soluble bridging protein Gas6, while αvβ3/5 integrins recognize PS through MFG-E8. Calreticulin, translocated to the apoptotic cell surface, provides an additional “eat-me” signal. Conversely, CD47 on the apoptotic cell surface engages SIRPα on the phagocyte membrane, transmitting an inhibitory “don’t-eat-me” signal that is attenuated during apoptosis. Downstream of receptor engagement, the small GTPase Rac1 is activated to drive cytoskeletal remodeling and phagocytic cup formation. The coordinated balance between pro-phagocytic (“eat-me”) and anti-phagocytic (“don’t-eat-me”) signals ultimately determines whether efferocytosis proceeds. PS, phosphatidylserine; MerTK, Mer tyrosine kinase; Gas6, growth arrest-specific 6; MFG-E8, milk fat globule-EGF factor 8; CD47, cluster of differentiation 47; SIRPα, signal regulatory protein alpha; Rac1, Rac family small GTPase 1.

In **Figure 1**, we illustrate the molecular “handshake” at the efferocytic synapse between an apoptotic cell and a phagocyte. Apoptotic cells recruit phagocytes by releasing “find-me” signals, a process initiated during early apoptosis. The primary “find-me” signals include nucleotides (ATP and UTP), lysophosphatidylcholine (LPC), and sphingosine-1-phosphate (S1P) (6, 7).

##### 2.1.1.1 Nucleotide signals

ATP and UTP represent classical “find-me” signals. Apoptotic cells release equimolar amounts of ATP and UTP through pannexin-1 channels in a caspase-dependent manner (15). These nucleotides act on monocytes and macrophages via the purinergic receptor P2Y2, promoting their migration toward apoptotic cells. *In vivo* mouse models demonstrate that depletion of nucleotides or inhibition of P2Y2 receptor function significantly impairs the clearance of apoptotic thymocytes (15).

Metabolites released during apoptosis function as tissue messengers. These metabolites encompass not only nucleotides but also various intracellular metabolites that collectively promote phagocyte recruitment and activation (16).

Extracellular ATP-hydrolyzing enzymes (such as CD39) regulate extracellular nucleotide levels by degrading ATP, thereby modulating the intensity and duration of “find-me” signals (17).

##### 2.1.1.2 Other “Find-Me” signals

In addition to nucleotides, LPC and S1P participate in phagocyte recruitment. These signaling molecules act through distinct receptor systems, forming complementary chemotactic gradients that ensure effective localization of phagocytes to apoptotic cells (7).

#### 2.1.2 Exposure of “Eat-Me” signals

##### 2.1.2.1 Phosphatidylserine exposure

PS externalization represents the most critical “eat-me” signal. In viable cells, PS is maintained in the inner leaflet of the plasma membrane by P4-type ATPases (flippases) such as ATP11A and ATP11C. During apoptosis, caspases inactivate flippases through cleavage and activate phosphatidylserine scramblases (primarily Xkr8), leading to rapid PS exposure on the cell surface (18, 19).

Xkr8 is a direct substrate of caspase-3; upon cleavage and activation, it mediates rapid PS externalization (20). Exposed PS is recognized by multiple receptor systems as an “eat-me” signal, including direct PS receptors (TIM-1/3/4, BAI1) and receptors that recognize PS via bridging proteins (MerTK via Gas6, αvβ3/5 integrins via MFG-E8) (21).

Notably, PS exposure is not exclusive to apoptotic cells. Under certain physiological conditions, viable cells may also expose PS, but this does not necessarily trigger phagocytosis (22). Therefore, PS exposure requires synergy with other signals to effectively trigger efferocytosis.

##### 2.1.2.2 Role of calreticulin

Calreticulin represents another important “eat-me” signal. During apoptosis or stress, calreticulin translocates from the endoplasmic reticulum to the cell surface, where it is recognized by the LRP1 receptor on phagocytes, thereby enhancing PS-mediated efferocytosis efficiency (23).

##### 2.1.2.3 Plasminogen-mediated eat-me signaling

Beyond phosphatidylserine and calreticulin, a distinct and clinically relevant eat-me signaling axis involves serum-derived plasminogen. During apoptosis and secondary necrosis, cell surface-expressed plasminogen activators—including tissue-type plasminogen activator (tPA) and urokinase (uPA) exposed on the apoptotic cell surface—convert circulating plasminogen into active plasmin (24). The resulting plasmin then acts as a soluble eat-me signal recognized by the mannose-6-phosphate/insulin-like growth factor 2 receptor (M6P/IGF2R, also known as the cation-independent mannose-6-phosphate receptor, CI-MPR), which is overexpressed on macrophage surfaces upon inflammatory activation (25). Plasminogen-induced efferocytosis via M6P/IGF2R has been demonstrated to significantly promote macrophage phagocytic clearance in murine *in vivo* models, and this axis has been further shown to be specifically triggered by signals derived from apoptotic cells themselves (26). This pathway is particularly relevant in the setting of chronic gastrointestinal inflammation, where secondary necrosis of uncleared apoptotic neutrophils generates abundant plasminogen-activating stimuli, and where potential dysregulation of M6P/IGF2R expression or plasminogen availability may contribute to impaired efferocytic clearance. The major efferocytosis receptors, their cognate eat-me ligands, bridging proteins, downstream signaling cascades, and disease-relevant alterations are summarized in **Table 1**.

**Table 1:** Summary of efferocytosis receptors, ligands, and bridging proteins.

Receptor	Type	Eat-me ligand	Bridging protein	Downstream signaling	Disease-relevant alterations	Key references
MerTK	TAM receptor tyrosine kinase	PS	Gas6, Protein S	PI3K-Akt; STAT1	Downregulated in IBD and NASH; ectodomain shedding mediated by ADAM17	(21, 34, 35, 64) (90)
TIM-4	Type I transmembrane protein	PS	—	DOCK180-Rac1	Reduced expression in IBD lamina propria macrophages	(21, 91).
αvβ3/αvβ5 integrins	Integrins	PS	MFG-E8 (lactadherin)	FAK-Src	MFG-E8 deficiency exacerbates inflammation in colitis models	(33, 36)
BAI1	GPCR	PS	—	ELMO-DOCK180-Rac1	Expressed on macrophages and microglia	(33, 36)
M6P/IGF2R	Mannose-6-phosphate receptor (CI-MPR)	Plasmin	Plasminogen	Downstream phagocytic facilitation signals	Overexpressed on macrophages following inflammatory activation	(24–26)
LRP1	Endocytic receptor	Calreticulin	—	PI3K	Expressed on both macrophages and hepatocytes	(23)
CD47 (*don’t-eat-me*)	Integrin-associated protein	SIRPα (inhibitory ligand)	—	SHP-1/SHP-2; non-muscle myosin IIA inhibition	Overexpressed during active IBD and in tumors	(27, 28, 65, 92, 93)
CD24 (*don’t-eat-me*)	GPI-anchored protein	Siglec-10 (inhibitory ligand)	—	ITIM-dependent pathway	Emerging immune evasion mechanism	(31)

BAI1, brain-specific angiogenesis inhibitor 1; CI-MPR, cation-independent mannose-6-phosphate receptor; DOCK180, dedicator of cytokinesis 180; ELMO, engulfment and cell motility protein; FAK, focal adhesion kinase; GPI, glycosylphosphatidylinositol; IBD, inflammatory bowel disease; ITIM, immunoreceptor tyrosine-based inhibitory motif; LRP1, low-density lipoprotein receptor-related protein 1; M6P/IGF2R, mannose-6-phosphate/insulin-like growth factor 2 receptor; MerTK, Mer tyrosine kinase; MFG-E8, milk fat globule-EGF factor 8; NASH, nonalcoholic steatohepatitis; PI3K, phosphatidylinositol 3-kinase; PS, phosphatidylserine; Rac1, ras-related C3 botulinum toxin substrate 1; SHP, Src homology region 2 domain-containing phosphatase; Siglec-10, sialic acid-binding immunoglobulin-type lectin 10; SIRPα, signal regulatory protein α; STAT1, signal transducer and activator of transcription 1; TAM, Tyro3/Axl/MerTK; TIM-4, T cell immunoglobulin and mucin domain-containing protein 4.

The *don’t-eat-me* signals CD47 and CD24 are included for direct mechanistic contrast with eat-me receptors; both function as inhibitory checkpoints suppressing efferocytosis rather than promoting it. M6P/IGF2R-mediated efferocytosis involves plasminogen activated at the apoptotic cell surface as the proximal eat-me signal, representing a pathway mechanistically distinct from phosphatidylserine-dependent recognition (24–26). Receptor shedding by ADAM17 (notably of MerTK) generates soluble decoy receptors that competitively inhibit membrane-bound receptor function and serves as a measurable biomarker of efferocytic dysfunction in NASH (90).

#### 2.1.3 Regulation of “Don’t-Eat-Me” signals

##### 2.1.3.1 CD47-SIRPα signaling axis

CD47 constitutes the principal “don’t-eat-me” signal, widely expressed on normal cell surfaces. Upon binding to SIRPα on phagocyte surfaces, CD47 recruits SHP phosphatases to inhibit phagocytic signaling (27). Normal cells avoid phagocytosis by maintaining high CD47 expression levels, whereas downregulation or conformational changes of CD47 during apoptosis attenuate this protective effect (28).

The CD47-SIRPα axis plays important roles in various pathological conditions. CD47 overexpression in tumor cells represents a critical mechanism of tumor immune evasion (29). CD47-blocking antibodies demonstrate enhanced phagocytosis and anti-tumor activity in preclinical studies (30).

##### 2.1.3.2 Other “Don’t-Eat-Me” signals

CD24-Siglec-10 represents a newly identified “don’t-eat-me” signaling pathway, wherein CD24 inhibits phagocytosis through binding to Siglec-10 on macrophage surfaces (31). PD-L1 also possesses “don’t-eat-me” function, inhibiting efferocytosis through engagement with PD-1 on phagocytes (32).

### 2.2 Phagocyte-mediated efferocytosis

#### 2.2.1 Macrophage efferocytosis mechanisms

**Figure 2:**
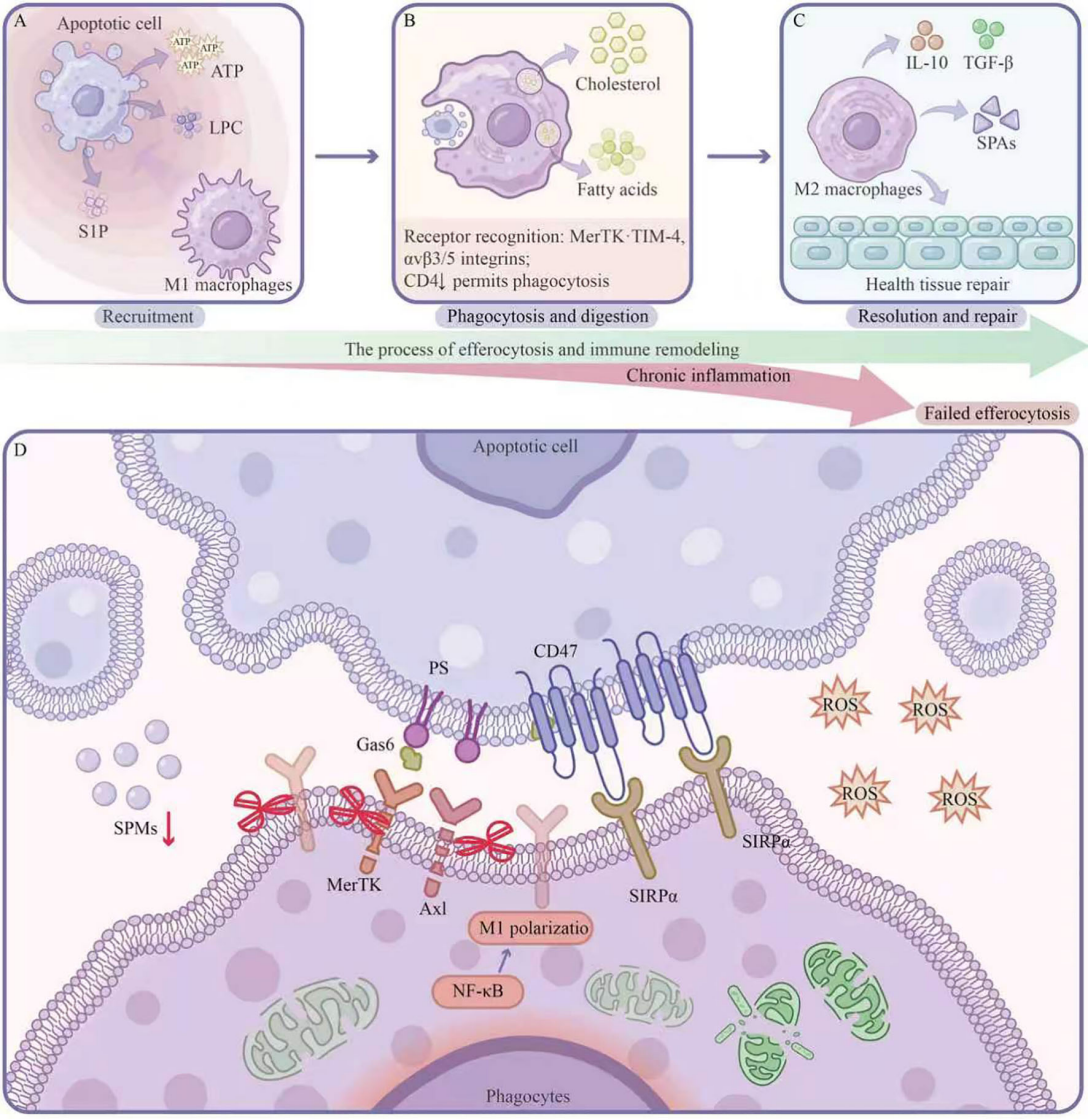
Sequential stages of successful efferocytosis and molecular mechanisms of failed efferocytosis in chronic inflammation. **(A–C)** The process of efferocytosis and immune remodeling. The efferocytic process proceeds through functionally distinct phases. **(A)** Recruitment: Apoptotic cells release “find-me” signals, including ATP, LPC, and S1P, which form chemotactic gradients that recruit M1-polarized macrophages to the site of cell death. **(B)** Phagocytosis and digestion: Macrophages engulf apoptotic cells and process their contents; liberated cholesterol and fatty acids serve as metabolic substrates that initiate intracellular reprogramming. **(C)** Resolution and repair: Following apoptotic cargo processing, macrophages convert to an M2 anti-inflammatory phenotype, secreting IL-10 and TGF-β, and producing SPMs that collectively promote healthy tissue repair. **(D)** Failed efferocytosis under chronic inflammation. In the chronically inflamed microenvironment, multiple converging defects impair clearance. On the phagocyte membrane, efferocytic receptors (MerTK, Axl) are downregulated or functionally inactivated (crossed symbols), and Gas6 availability is diminished. Sustained CD47-SIRPα engagement transmits dominant inhibitory signals. Within the phagocyte, NF-κB activation drives M1 polarization, while mitochondrial dysfunction compromises the metabolic support required for engulfment. Extracellularly, elevated ROS oxidatively modify PS and receptors, reducing binding affinity, while decreased SPM levels (SPMs↓) deprive the system of pro-resolving amplification signals, establishing a self-reinforcing cycle of sustained inflammation. ATP, adenosine triphosphate; LPC, lysophosphatidylcholine; S1P, sphingosine-1-phosphate; IL-10, interleukin-10; TGF-β, transforming growth factor-beta; SPMs, specialized pro-resolving mediators; ROS, reactive oxygen species; NF-κB, nuclear factor kappa B.

Macrophages represent the primary effector cells executing efferocytosis, and this phagocytic capacity is not restricted to any single polarization state. **Figures 2A–C** illustrates the continuum of apoptotic cell clearance, encompassing the sequential stages of efferocytosis and the ensuing phenotypic transition in macrophages. Unpolarized M0 macrophages perform efferocytosis efficiently under basal conditions, and even M1-polarized (pro-inflammatory) macrophages retain substantial phagocytic capacity—though with lower efficiency and critically different downstream signaling consequences compared with M2 macrophages (33). What macrophage polarization fundamentally determines is not whether efferocytosis occurs, but rather the nature of the post-efferocytosis transcriptional program: M2-driven efferocytosis is tightly coupled to a pro-resolving response (IL-10, TGF-β secretion; SPM biosynthesis) that actively terminates inflammation, whereas M1-driven efferocytosis may lack this coupling, leaving the inflammatory cascade active despite ongoing apoptotic cell clearance. In the setting of chronic gastrointestinal diseases—where M1 macrophages predominate in inflamed tissues—this mechanistic distinction is clinically pivotal: it explains why histological evidence of phagocytic activity does not always correlate with inflammation resolution, and identifies the restoration of pro-resolving efferocytosis coupling (not merely efferocytic frequency) as the appropriate therapeutic objective.

MerTK, a member of the TAM receptor family, plays a central role in efferocytosis. MerTK not only mediates recognition and engulfment of apoptotic cells but also triggers downstream anti-inflammatory and pro-resolving signaling pathways (34). Following MerTK activation, the PI3K-Akt pathway promotes macrophage polarization toward the M2 anti-inflammatory phenotype (35).

Upon efferocytosis receptor activation, the DOCK180-ELMO-Rac1 signaling pathway drives actin polymerization and phagocytic cup formation (36). Apoptotic cells, once engulfed, form specialized phagosomes (efferosomes) characterized by lower acidification levels, favoring anti-inflammatory signal release (37).

#### 2.2.2 Role of non-professional phagocytes

Beyond macrophages, epithelial cells and dendritic cells possess certain efferocytosis capabilities. Airway epithelial cells can clear apoptotic neutrophils through efferocytosis, which is crucial for respiratory inflammation resolution (38). However, epithelial cell efferocytosis efficiency is substantially lower than that of professional phagocytes (39).

Dendritic cells play dual roles in efferocytosis. Under homeostatic conditions, dendritic cell efferocytosis of apoptotic cells induces immune tolerance; conversely, under inflammatory contexts, it may trigger immune responses (40, 41). Intestinal dendritic cells play important roles in maintaining intestinal immune homeostasis (42).

### 2.3 Immune regulation following efferocytosis

#### 2.3.1 Macrophage phenotype transition

Efferocytosis triggers macrophage transition from pro-inflammatory to anti-inflammatory phenotypes. Following apoptotic cell engulfment, macrophages undergo reprogramming through multiple signaling pathways: MerTK signaling inhibits NF-κB and MAPK pathways; apoptotic cell-derived lipids activate PPARγ and LXR nuclear receptors; phagosomal metabolic reprogramming promotes oxidative phosphorylation and IL-10 production (43, 44).

Regulatory T cells (Tregs) enhance macrophage efferocytosis capacity and anti-inflammatory function. Tregs enhance macrophage apoptotic cell clearance efficiency through IL-10 secretion and direct cell contact, thereby promoting inflammation resolution (45).

Single-cell sequencing studies reveal the emergence of “resolution-associated macrophage” subpopulations with distinct transcriptional signatures during inflammation resolution, characterized by high expression of efferocytosis receptors and anti-inflammatory factors (46).

#### 2.3.2 Anti-inflammatory cytokine release

##### 2.3.2.1 Critical role of IL-10

IL-10 represents the most important anti-inflammatory cytokine induced by efferocytosis. IL-10 exerts anti-inflammatory effects through multiple mechanisms: suppressing pro-inflammatory cytokine production, downregulating antigen presentation molecule expression, and inducing Treg differentiation (47, 48).

In the intestine, IL-10R (IL-10 receptor) signaling is crucial for maintaining immune homeostasis. IL-10 receptor signaling in innate immune cells regulates mucosal immune tolerance and anti-inflammatory macrophage function (10). Intestinal CX3CR1+ macrophages represent the primary source of efferocytosis-dependent IL-10 (49).

##### 2.3.2.2 Role of TGF-β

TGF-β represents another important efferocytosis-induced cytokine. TGF-β inhibits pro-inflammatory activity of T cells and macrophages while promoting Treg differentiation (50). However, during chronic inflammation, sustained TGF-β signaling may promote fibrosis (51).

#### 2.3.3 Initiation of inflammation resolution programs

##### 2.3.3.1 Specialized pro-resolving mediators

Efferocytosis promotes SPM biosynthesis. SPMs encompass lipoxins, resolvins, protectins, and maresins, derived from enzymatic metabolism of omega-3 and omega-6 polyunsaturated fatty acids (8, 52).

Following apoptotic cell engulfment, macrophages upregulate lipoxygenase expression, converting apoptotic cell-derived fatty acid substrates into SPMs (53). These mediators exert pro-resolving actions through specific receptors: promoting neutrophil apoptosis and reverse migration, enhancing macrophage efferocytosis capacity, and suppressing pro-inflammatory mediator production (54).

##### 2.3.3.2 Clinical significance of inflammation resolution

Decreased SPM levels in inflammatory diseases correlate with disease activity. Supplementation with exogenous SPMs can ameliorate experimental colitis and other inflammatory models (55, 56). However, chemical instability of SPMs and optimal delivery strategies remain challenges for clinical translation (57).

Inflammation resolution encompasses not merely termination of pro-inflammatory signals but represents an active biological program, including accelerated neutrophil apoptosis, macrophage phenotype transition, Treg expansion, and tissue repair initiation (58). “Resolution deficiency” is considered a core pathological mechanism of chronic inflammatory diseases (59).

## 3 Impaired neutrophil efferocytosis in chronic inflammatory digestive diseases

The hallmark pathological feature of chronic inflammatory digestive diseases is persistent tissue damage resulting from dysregulated inflammation resolution. Defective neutrophil efferocytosis serves as a key driver in the pathogenesis and progression of these disorders, involving abnormalities in efferocytic receptor systems, signaling pathway disruptions, and microenvironmental interference at multiple levels. **Figure 2D** illustrates the molecular-level mechanism of failed clearance between apoptotic cells (above) and phagocytes (below).

### 3.1 Inflammatory bowel disease

Inflammatory bowel disease, encompassing Crohn’s disease (CD) and ulcerative colitis (UC), is characterized by chronic relapsing intestinal inflammation. Neutrophil infiltration represents a hallmark pathological feature during active IBD, while impaired clearance of apoptotic neutrophils constitutes a core mechanism underlying persistent inflammation.

#### 3.1.1 Efferocytic defects in IBD

Specifically, defects in interleukin-10 receptor (IL-10R) signaling—not merely reduced IL-10 production—in intestinal macrophages impair their conversion to anti-inflammatory phenotypes, subsequently reducing their capacity to phagocytose apoptotic neutrophils, a deficiency closely associated with disease activity (10). Studies using myeloid-specific IL-10R conditional knockout mice (LysM-Cre × IL10Rb^fl/fl^) have demonstrated that selective disruption of the IL-10R→STAT3 signaling axis in innate immune cells leads to severe spontaneous colitis, accompanied by profound failure to generate anti-inflammatory macrophages and near-complete loss of efferocytic activity in lamina propria macrophages (60). These conditional knockout data establish IL-10R→STAT3 as a non-redundant axis governing macrophage efferocytic competence in the intestinal microenvironment, and distinguish receptor-level signaling failure from mere IL-10 deficiency as the proximal mechanism of efferocytic dysfunction in IBD.

Apoptotic neutrophils that fail to be promptly cleared in inflamed intestinal tissues undergo secondary necrosis, releasing damage-associated molecular patterns (DAMPs). These DAMPs activate immune cells through pattern recognition receptors, establishing inflammatory amplification loops (9). Neutrophils also release extracellular traps (NETs), whose DNA-protein complexes exert pro-inflammatory effects and exacerbate tissue damage through complement system activation and thrombus induction (61). NETs accumulate extensively in the intestinal mucosa of IBD patients, correlating positively with disease severity.

Single-cell sequencing technologies have revealed heterogeneous alterations in intestinal macrophages of IBD patients. A pro-inflammatory macrophage subset emerges in diseased tissues, characterized by high expression of pro-inflammatory cytokines such as IL-1β and TNF-α, but significantly reduced expression of efferocytosis-related genes (including MERTK, TIMD4, and MFGE8) (62). The imbalance between tissue-resident macrophages and inflammatory monocyte-derived macrophages constitutes an important cellular basis for efferocytic deficiency (63). The former typically possess robust efferocytic capacity and tissue repair functions, whereas the latter, despite increased numbers, exhibit lower efferocytic efficiency.

#### 3.1.2 Molecular mechanisms

Impaired “eat-me” signal recognition systems represent a core mechanism underlying efferocytic defects in IBD. The TAM receptor signaling system plays a crucial role in regulating efferocytosis, with bridging proteins Gas6 and Protein S mediating the binding between apoptotic cell phosphatidylserine (PS) and receptors (64). In IBD patients, reduced expression of these bridging proteins leads to decreased macrophage recognition efficiency of apoptotic cells. Concurrently, downregulation of another important bridging protein, MFG-E8, further weakens the integrin αvβ5-mediated efferocytic pathway.

Aberrant expression of the “don’t-eat-me” signal CD47 represents another key mechanism. Inflammatory cytokines TNF-α and IFN-γ upregulate CD47 transcription through NF-κB and STAT1 pathways, with CD47 binding to macrophage surface SIRPα generating inhibitory signals that impede efferocytosis (65). Experimental studies demonstrate that blocking the CD47-SIRPα signaling axis promotes apoptotic cell clearance and reduces tissue inflammation (66). This finding provides a novel therapeutic target for IBD.

Oxidative stress in the inflammatory microenvironment can modify apoptotic cell surface PS, causing conformational changes or oxidation that reduce binding affinity to efferocytic receptors (67). Aberrant activation of matrix metalloproteinases can cleave the extracellular domains of efferocytic receptors, resulting in receptor dysfunction (68). Additionally, gut microbiota dysbiosis participates in regulating efferocytic function, with certain pathogenic bacteria capable of directly suppressing macrophage activity or affecting efferocytic efficiency through metabolic products (69).

#### 3.1.3 Clinical relevance

Efferocytic function correlates with IBD treatment response. Macrophage phenotype and functional status may influence patient responsiveness to biological agents, with patients possessing stronger efferocytic capacity often showing better responses to anti-TNF-α therapy. Genome-wide association studies have identified multiple immune-related gene variants associated with IBD risk, some involving integrin signaling and cellular phagocytic functions (70). These genetic findings provide additional support for the importance of efferocytic defects in IBD pathogenesis. Tissue-level SPM profiling provides additional support for the efferocytosis-resolution coupling in IBD. Biopsies from ulcerative colitis patients show significantly elevated mucosal Annexin A1 and lipoxin A4 (LXA4) compared with non-inflamed controls, suggesting a compensatory upregulation of endogenous pro-resolving signaling that nonetheless proves insufficient to overcome efferocytic deficiency during active flares (55). Notably, exogenous LXA4 and its stable analogs restore macrophage efferocytic capacity in murine colitis models via the ALX/FPR2 receptor, and concurrent blockade of 5-LOX—the rate-limiting enzyme in lipoxin biosynthesis—exacerbates mucosal neutrophil accumulation, underscoring the LXA4-ALX/FPR2 axis as both a pathophysiologically relevant and therapeutically actionable target in IBD.

### 3.2 Chronic gastritis and peptic ulcer disease

Helicobacter pylori (H. pylori) infection represents the primary etiology of chronic gastritis and peptic ulcer disease. Neutrophil infiltration is a typical feature of infection-associated gastric mucosal lesions, with efferocytic dysfunction participating in disease chronification and affecting mucosal repair processes.

#### 3.2.1 Effects of H. pylori on efferocytosis

H. pylori interferes with gastric mucosal immune function through multiple mechanisms. Bacterial virulence factors VacA and CagA directly affect macrophage phagocytic and degradative functions, with VacA inhibiting phagosome-lysosome fusion and CagA interfering with efferocytic processes through activation of host cell signaling pathways (71). This “incomplete efferocytosis” paradoxically exacerbates inflammatory responses, as inadequately degraded apoptotic cell contents can leak into the cytoplasm and activate inflammatory signals.

Infection-induced inflammatory cytokines and chemokines, while recruiting neutrophils, may also affect macrophage efferocytic function. Overexpression of chemokines such as IL-8 not only promotes neutrophil infiltration but can also inhibit macrophage conversion to anti-inflammatory phenotypes through autocrine/paracrine mechanisms (72). Upregulation of CD47 expression in infected tissues inhibits apoptotic cell clearance through “don’t-eat-me” signals (73).

Following H. pylori eradication, gastric mucosal inflammation can gradually resolve, though recovery extent varies with infection duration, strain virulence, and host factors (74). Mucosal atrophy and metaplasia resulting from prolonged infection may be incompletely reversible, underscoring the importance of early eradication therapy.

#### 3.2.2 Gastric mucosal repair and efferocytosis

Efferocytosis plays an important role in gastric mucosal damage repair. Macrophages not only clear necrotic cell debris but also promote epithelial regeneration and angiogenesis through secretion of epidermal growth factor (EGF), vascular endothelial growth factor (VEGF), and other factors (75). The gastric mucosa contains repair-associated cell subsets expressing trefoil factor peptides (TFF), which synergize with macrophages to promote mucosal integrity restoration (76).

In chronic gastritis, persistent inflammation and oxidative stress impair macrophage function. Accumulation of apoptotic cells in tissues correlates with reduced macrophage numbers and impaired function, establishing a vicious cycle of “inflammation-efferocytic deficiency-persistent inflammation” (77). Breaking this cycle requires simultaneously addressing the etiology (such as H. pylori eradication) and modulating immune responses.

### 3.3 Nonalcoholic fatty liver disease

**Figure 3:**
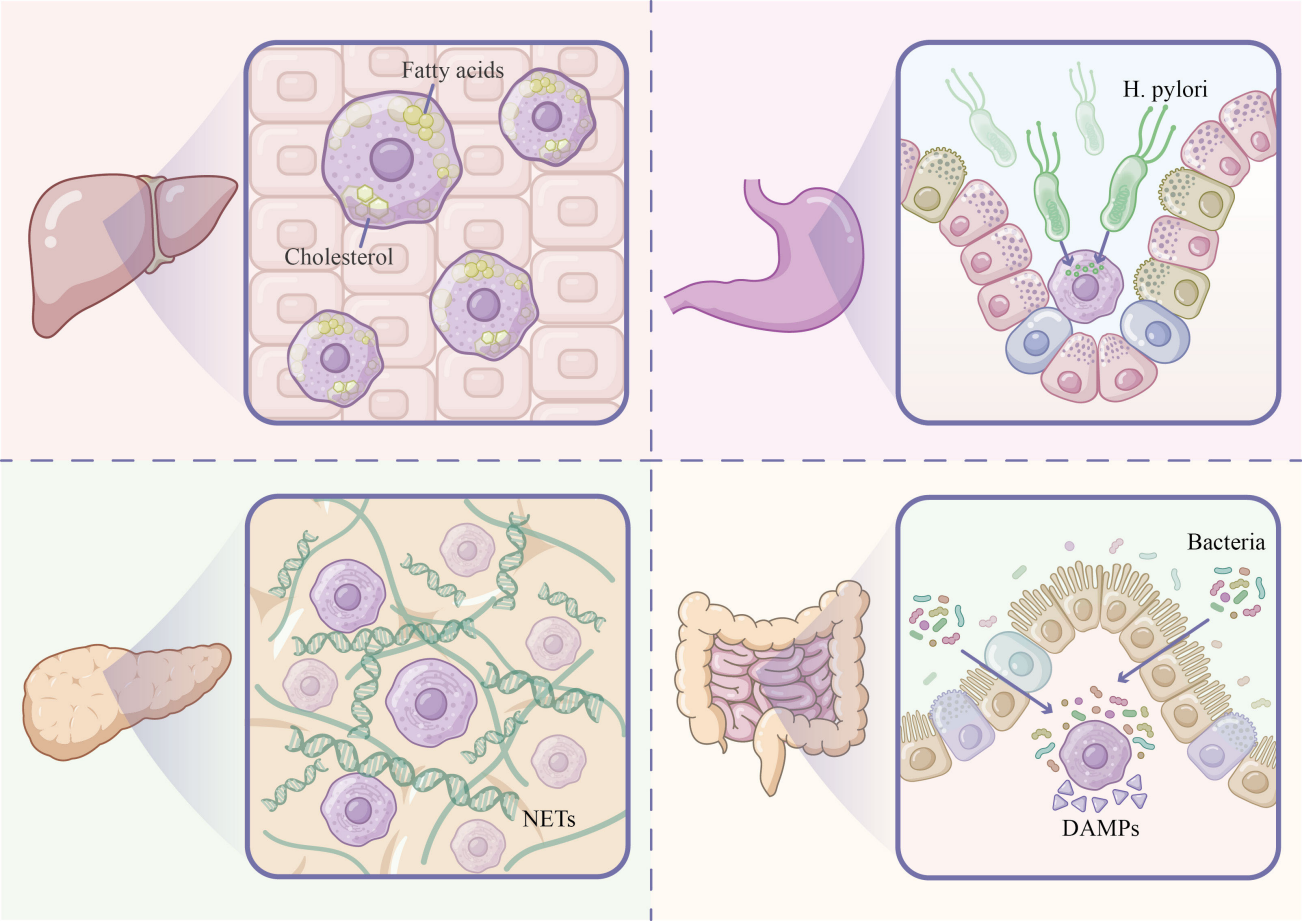
Organ-specific manifestations of efferocytic dysfunction across chronic inflammatory gastrointestinal diseases. The figure illustrates four distinct disease contexts in which impaired efferocytosis contributes to pathology. (Upper left) Liver - NAFLD/NASH: Lipid overload within Kupffer cells, characterized by intracellular accumulation of free fatty acids and cholesterol crystals, impairs efferocytic receptor function and promotes inflammatory responses. (Upper right) Stomach - Chronic gastritis: *H. pylori* infection disrupts gastric mucosal immune homeostasis; bacterial virulence factors interfere with macrophage phagocytic and degradative capacity, contributing to persistent mucosal inflammation. (Lower left) Pancreas - Acute/Chronic pancreatitis: Excessive NET formation leads to the accumulation of DNA-protein complexes in pancreatic tissue; impaired NET clearance amplifies acinar cell damage and drives disease chronification. (Lower right) Intestine - IBD: Secondary necrosis of uncleared apoptotic cells releases DAMPs, which activate mucosal immune cells via pattern recognition receptors and are further compounded by dysbiotic bacteria breaching the epithelial barrier, perpetuating the inflammatory cycle. NAFLD, non-alcoholic fatty liver disease; NASH, non-alcoholic steatohepatitis; H. pylori, Helicobacter pylori; NETs, neutrophil extracellular traps; IBD, inflammatory bowel disease; DAMPs, damage-associated molecular patterns.

Nonalcoholic fatty liver disease (NAFLD) and its progressive form, nonalcoholic steatohepatitis (NASH), represent common chronic liver diseases. Neutrophil infiltration and efferocytic defects play crucial roles in NASH pathogenesis and progression, closely associating with hepatic fibrosis and cirrhosis development. **Figure 3** illustrates the key determinants and their corresponding molecular mechanisms.

#### 3.3.1 Neutrophils in NASH

The degree of hepatic neutrophil infiltration in NASH patients correlates with disease severity. Neutrophil-derived myeloperoxidase (MPO), elastase, and reactive oxygen species (ROS) can directly damage hepatocytes, inducing lipid peroxidation and mitochondrial dysfunction (12). Peripheral blood neutrophils exhibit abnormal activation phenotypes with delayed apoptotic programs, resulting in excessive accumulation in hepatic tissue.

Accumulation of apoptotic and necrotic neutrophils in hepatic tissue associates with fibrosis progression. Neutrophil-released serine proteases (including elastase, cathepsin G, and proteinase 3) can directly activate hepatic stellate cells, promoting their transformation to myofibroblast phenotypes (78). This process drives hepatic fibrosis progression through TGF-β signaling activation and increased extracellular matrix deposition. Neutrophil-released NETs also participate in fibrotic processes, with histones and DNA-protein complexes in NETs serving as damage signals that activate hepatic stellate cells.

#### 3.3.2 Kupffer cell dysfunction

Kupffer cells, the liver’s resident macrophages comprising 20-30% of hepatic non-parenchymal cells, are the primary effector cells executing hepatic efferocytic function. In NASH, expression of efferocytic receptors MerTK and CD36 on Kupffer cells decreases, with function significantly impaired (79). Lipid overload, particularly saturated fatty acids and oxidized low-density lipoprotein (oxLDL), affects Kupffer cell metabolic and functional states through endoplasmic reticulum stress and mitochondrial dysfunction (80).

Cholesterol crystal accumulation within Kupffer cells can activate the NLRP3 inflammasome, leading to IL-1β and IL-18 maturation and secretion (81). These pro-inflammatory cytokines not only exacerbate local inflammation but also further suppress Kupffer cell efferocytic function through autocrine/paracrine mechanisms, establishing an inflammation-efferocytic deficiency vicious cycle. Additionally, “foamy” Kupffer cells resulting from lipid overload, while maintaining some phagocytic capacity, exhibit significantly reduced specificity for apoptotic cell recognition and processing efficiency.

Single-cell transcriptomic analysis reveals significant alterations in macrophage subset composition in NASH liver. The proportion of resident Kupffer cells (expressing high levels of CLEC4F and TIMD4) with normal efferocytic function and tissue repair capacity decreases, while pro-inflammatory monocyte-derived macrophages (expressing high levels of Ly6C and CCR2) increase (82). This alteration in cellular composition represents an important cellular basis for overall efferocytic function decline in NASH.

#### 3.3.3 Efferocytic deficiency and NASH progression

Experimental studies demonstrate that enhancing efferocytic function can reduce hepatic inflammation and fibrosis. Specialized pro-resolving mediators (SPMs) such as resolvins and protectins exert anti-inflammatory and anti-fibrotic effects by activating efferocytic receptors, promoting macrophage M2 polarization, and enhancing apoptotic cell clearance (54). In NASH animal models, exogenous SPM supplementation or enhanced endogenous SPM generation can improve disease progression. Among the SPMs studied in NASH, Resolvin D1 (RvD1) has received particular mechanistic attention. Signaling through its cognate receptors ALX/FPR2 and GPR32 on Kupffer cells and recruited hepatic macrophages, RvD1 dose-dependently enhances the efferocytosis of lipoapoptotic hepatocytes in both palmitate-stimulated *in vitro* macrophage systems and diet-induced murine NASH models (54). In human biopsy cohorts, hepatic RvD1 concentrations are inversely correlated with histological NASH activity scores, establishing RvD1 deficiency as a clinically meaningful component of the resolution failure driving NASH progression—and providing a direct mechanistic rationale for omega-3 PUFA supplementation strategies discussed in Section 4.6.

Clinical studies show that expression levels of efferocytosis-related molecules correlate with NASH severity. Elevated serum soluble MerTK (sMerTK) levels positively correlate with hepatic fibrosis degree, potentially serving as a non-invasive biomarker of disease progression (83). sMerTK is generated from membrane-bound MerTK through cleavage by metalloproteinases such as ADAM17, with its elevation reflecting impaired efferocytic receptor function in tissues.

### 3.4 Acute pancreatitis and its chronification

Acute pancreatitis (AP) is a common gastrointestinal emergency characterized by acute pancreatic inflammation. Neutrophils are the primary effector cells in early AP inflammatory responses, with their impaired clearance closely relating to disease severity and risk of progression to chronic pancreatitis (CP).

#### 3.4.1 Neutrophils in acute pancreatitis

Inflammatory mediators released after pancreatic acinar cell damage (including IL-6, IL-8, TNF-α, and chemokines CXCL1/2) recruit abundant neutrophils to pancreatic tissue (13). Early neutrophil infiltration serves protective functions, limiting secondary infection through debris phagocytosis and antimicrobial peptide secretion. However, excessive or sustained neutrophil activation becomes a major driver of tissue damage.

In mild AP, neutrophils undergo programmed apoptosis 48–72 hours post-inflammation peak and are effectively cleared, with subsequent macrophage-mediated efferocytosis promoting inflammation resolution and tissue repair (84). In severe AP, neutrophil apoptotic programs are delayed while efferocytic function is impaired, resulting in persistent activated neutrophils. These cells continuously release elastase, cathepsins, and ROS, exacerbating pancreatic necrosis and systemic inflammatory response syndrome (SIRS). (see **Figure 3**).

NET levels are significantly elevated in pancreatic tissue and serum of severe AP patients. NETs exacerbate disease through multiple mechanisms: direct damage to acinar cells and vascular endothelial cells; activation of trypsinogen leading to abnormal pancreatic enzyme activation; induction of microthrombosis exacerbating tissue ischemia (85). NET clearance also depends on macrophage efferocytic function, with efferocytic dysfunction leading to excessive NET accumulation, forming a vicious cycle of “neutrophil activation-NET formation-tissue damage-inflammation amplification”.

#### 3.4.2 Pancreatic macrophage functional changes

Pancreatic resident macrophages efficiently execute efferocytic functions under homeostatic conditions, clearing daily naturally apoptotic acinar cells and infiltrating immune cells to maintain tissue homeostasis (86). During AP onset, the pancreatic microenvironment undergoes dramatic changes, significantly affecting macrophage function.

In the acute phase of AP (1–3 days post-onset), macrophages predominantly exhibit M1 pro-inflammatory phenotypes, highly expressing iNOS, IL-12, and TNF-α, with reduced efferocytic capacity. During recovery (5–7 days post-onset), under the influence of Th2 cytokines such as IL-4 and IL-13, some macrophages convert to M2 anti-inflammatory phenotypes, expressing arginase-1 (Arg-1), CD206, and efferocytic receptors, with gradually recovering efferocytic function (87). However, in severe AP patients, this phenotypic conversion is severely delayed or absent, associating with increased risk of disease chronification.

Multiple factors in the AP pancreatic microenvironment suppress macrophage efferocytic function. Abnormally activated pancreatic enzymes (particularly trypsin and elastase) can cleave macrophage surface efferocytic receptors and integrins; locally high concentrations of free fatty acids (released from adipose tissue necrosis) and calcium ions interfere with phagosome formation and membrane fusion processes; persistently high levels of pro-inflammatory cytokines (IL-1β, TNF-α, IFN-γ) maintain macrophages in pro-inflammatory states, inhibiting their conversion to efferocytically active phenotypes (88).

#### 3.4.3 Efferocytic deficiency and chronic pancreatitis

Recurrent AP represents the primary risk factor for CP development. Efferocytic dysfunction plays a crucial role in this chronification process. Characteristic pathological changes of CP include acinar atrophy, ductal dilation, islet destruction, and extensive fibrosis—changes partially stemming from persistent low-grade inflammation and cellular debris accumulation.

Abundant apoptotic cell debris and secondary necrotic products (including HMGB1, S100 proteins, and ATP) persist in pancreatic tissues of CP patients. These uncleared cellular remnants continuously stimulate fibrotic responses through TLR and RAGE receptors (89). Pancreatic stellate cells (PSCs) become persistently activated under stimulation by these damage signals, producing abundant extracellular matrix and driving progressive fibrosis. Enhancing efferocytic function may delay or prevent AP-to-CP progression by promoting inflammation resolution and reducing damage signals.

### 3.5 Common pathological mechanisms

Despite diverse etiologies and affected organs in the aforementioned diseases, defective efferocytosis serves as a common pathological mechanism involving similar molecular targets, signaling pathways, and microenvironmental factors. Identifying these shared features holds significant importance for developing broad-spectrum therapeutic strategies for chronic inflammatory digestive diseases.

#### 3.5.1 Efferocytic receptor downregulation

Downregulation of efferocytic receptors (such as MerTK, Axl, Tyro3, TIM-4, BAI1) represents a common feature across various chronic digestive inflammatory conditions. The TAM receptor family plays a central role in regulating efferocytosis, with its expression and function subject to multi-level regulation (64).

Pro-inflammatory cytokines suppress receptor expression through multiple pathways: TNF-α and IL-1β activate NF-κB pathways, inhibiting MERTK gene promoter activity; IFN-γ induces inhibitory microRNA expression (such as miR-21, miR-181) through STAT1 signaling—these microRNAs target MERTK mRNA for degradation; persistent oxidative stress accelerates receptor protein degradation through protein oxidative modification.

Ectodomain shedding represents another important mechanism. ADAM family metalloproteinases (particularly ADAM10 and ADAM17) can cleave membrane-bound MerTK, generating soluble MerTK (sMerTK) (90). sMerTK not only loses efferocytosis-mediating function but also acts as a “decoy receptor” competitively binding the ligand Gas6, further inhibiting residual membrane receptor activation. Elevated sMerTK levels are detected in serum and tissue fluids of patients with various chronic digestive inflammatory conditions.

Epigenetic modifications participate in regulating long-term expression changes in efferocytic receptor genes. Hypermethylation of CpG islands in the TIM-4 gene promoter region associates with its transcriptional repression—this epigenetic “imprint” may explain chronic inflammation persistence: even after acute inflammatory triggers are removed, efferocytic function remains difficult to fully restore (91). Histone modifications (such as H3K27me3 and H3K9me3) also participate in long-term efferocytic gene suppression.

#### 3.5.2 CD47-SIRPα axis aberrations

CD47 overexpression represents the primary mechanism of enhanced “don’t-eat-me” signals, universally present in multiple chronic inflammatory conditions. Multiple factors in the inflammatory microenvironment synergistically upregulate CD47 expression (27):

Hypoxia-inducible factor-1α (HIF-1α) directly binds hypoxia response elements (HRE) in the CD47 gene promoter region under tissue ischemia-hypoxia conditions, enhancing transcription. Diseased tissues in IBD, NASH, and pancreatitis all exhibit local hypoxia, making HIF-1α activation an important mechanism of CD47 upregulation.

NF-κB responds to TNF-α and IL-1β signals, enhancing transcription through binding to κB sites in the CD47 promoter region. Some microbial products such as lipopolysaccharide (LPS) also promote CD47 expression through TLR4-NF-κB signaling.

Targeting the CD47-SIRPα axis can enhance efferocytic function. Anti-CD47 monoclonal antibodies have demonstrated efficacy in tumor therapy by enhancing macrophage phagocytosis of tumor cells (92). In inflammatory diseases, CD47 blockade strategies also show potential therapeutic value. However, systemic CD47 blockade may cause increased red blood cell and platelet clearance, inducing anemia and thrombocytopenia adverse effects (93). Therefore, developing tissue-specific, inflammatory cell-specific, or apoptotic cell-specific CD47 targeting strategies, or employing low-dose intermittent dosing regimens, may maintain efficacy while reducing adverse effects.

#### 3.5.3 Macrophage polarization defects

Macrophage conversion to anti-inflammatory M2 and pro-resolution phenotypes represents an important prerequisite for efficient efferocytosis. Chronic inflammatory tissues universally lack resolution-associated macrophage subsets with high efferocytic capacity (94). This polarization defect involves abnormalities at multiple transcriptional and metabolic regulatory nodes.

Nuclear receptors PPARγ and LXR (liver X receptors) are core transcription factors for M2 polarization and efferocytic gene expression. PPARγ enhances transcription through direct binding to PPRE (PPAR response elements) in promoter regions of genes such as MERTK, CD36, and ABCA1, while simultaneously suppressing NF-κB activity to reduce pro-inflammatory cytokine production. LXR maintains macrophage membrane fluidity and phagocytic function by regulating lipid metabolism and cholesterol efflux. In chronic inflammation, both nuclear receptor expression and activity are reduced (94).

Conversely, sustained activation of NF-κB and STAT1 maintains macrophages in pro-inflammatory M1 phenotypes. Chronic inflammation-induced epigenetic changes, including increased inhibitory histone modifications (H3K27me3, H3K9me3) and decreased activating modifications (H3K4me3, H3K27ac) at efferocytic gene promoter regions, may “lock” macrophages in pro-inflammatory states (95). Long non-coding RNAs (lncRNAs) and microRNAs also participate in this epigenetic regulatory network.

#### 3.5.4 Specialized pro-resolving mediator deficiency

Specialized pro-resolving mediators (SPMs) are not only effector molecules in active inflammation resolution but can also directly enhance efferocytic function. The SPM family includes lipoxins, resolvins, protectins, and maresins, generated from omega-3 and omega-6 polyunsaturated fatty acids (PUFAs) through lipoxygenase pathways (8).

SPMs enhance efferocytosis through multiple mechanisms: upregulating macrophage MerTK and LXR expression; promoting macrophage M2 phenotype polarization; enhancing phagosome-lysosome fusion; facilitating intracellular degradation of phagocytosed apoptotic cells. For example, resolvin D1 (RvD1) enhances human monocyte-derived macrophage efferocytic capacity 2–3 fold by activating its receptors ALX/FPR2 and GPR32 (96).

SPM levels are significantly reduced in tissues of patients with chronic digestive inflammatory conditions, relating to multiple factors:

Substrate insufficiency: Modern diets contain inadequate omega-3 PUFAs (such as EPA and DHA) with excess omega-6 PUFAs, resulting in imbalanced omega-6/omega-3 ratios. Some patients also have fatty acid metabolic enzyme deficiencies.

Reduced synthetic enzyme activity: Pro-inflammatory cytokines in inflammatory environments suppress 12/15-lipoxygenase (12/15-LOX) and epoxide hydrolase expression—key rate-limiting enzymes in SPM biosynthesis. Oxidative stress can also inactivate these enzymes through oxidative modification.

Accelerated degradation: Oxidative environments in inflammatory tissues cause excessive SPM oxidation and inactivation. Additionally, elevated levels of pro-inflammatory lipid mediators (such as leukotrienes and prostaglandins) competitively bind receptors with SPMs.

These factors collectively produce SPM deficiency, establishing a self-reinforcing pathological cycle: efferocytic deficiency → SPM reduction → persistent inflammation → further efferocytic impairment. Breaking this cycle can be achieved by supplementing exogenous SPMs or by enhancing endogenous SPM biosynthesis through omega-3 PUFA supplementation.

#### 3.5.5 Metabolic reprogramming

Recent research has revealed that macrophage metabolic patterns profoundly influence their functional phenotypes, including efferocytic capacity. Efficient efferocytosis requires macrophages to maintain oxidative phosphorylation (OXPHOS)-predominant energy metabolism, which provides sustained stable ATP supply supporting the energy-intensive phagocytic and degradative processes (97).

However, chronic inflammatory microenvironments induce macrophage metabolic reprogramming from OXPHOS toward glycolysis (resembling the Warburg effect). Although glycolysis can rapidly generate ATP supporting pro-inflammatory functions (such as pro-inflammatory cytokine synthesis and ROS production), it disadvantages efferocytosis. Glycolytic macrophages have impaired mitochondrial function with reduced fatty acid oxidation (FAO), yet FAO-generated acetyl-CoA is crucial for maintaining phagosome membrane fluidity and lysosomal function.

The AMPK-mTOR signaling axis represents a key node connecting metabolic state and immune function (98), AMPK senses cellular energy status, with its activation promoting OXPHOS and FAO while upregulating PGC-1α (the master transcriptional regulator of mitochondrial biogenesis) and efferocytic receptor expression. Conversely, excessive mTORC1 activation promotes glycolysis and lipid synthesis, maintaining the pro-inflammatory M1 phenotype.

In multiple digestive inflammatory models, pharmacological AMPK activation (such as with metformin or AICAR) or mTORC1 inhibition (such as with rapamycin) can improve macrophage efferocytic function and promote inflammation resolution (99). This suggests metabolic intervention may represent a novel strategy for enhancing efferocytosis and treating chronic inflammation. Notably, availability of different metabolic substrates also affects macrophage phenotype: high glucose promotes glycolysis and M1 polarization, while glutamine and fatty acids favor OXPHOS and M2 polarization. Although the etiologies and affected organs of these four diseases differ substantially, the shared and disease-specific efferocytic defect mechanisms, representative experimental evidence, and pathological consequences are systematically compared in **Table 2**.

**Table 2:** Comparison of efferocytosis defects across four chronic inflammatory gastrointestinal diseases.

Disease	Core efferocytosis defects	Key molecular mechanisms	Representative experimental/clinical evidence	Pathological consequences of defective efferocytosis
IBD (UC/CD)	↓MerTK/TIM-4/MFG-E8; ↑CD47; IL-10R signaling failure	NF-κB-driven CD47 upregulation; IL-10R→STAT3 axis defect; ADAM17-mediated MerTK shedding	scRNA-seq identification of efferocytosis-impaired macrophage subpopulations (62, 63); myeloid-specific IL-10R conditional knockout mice (10)	Secondary necrosis of apoptotic cells; NET accumulation; mucosal barrier disruption; perpetuation of chronic inflammation
Chronic gastritis	*H. pylori* CagA suppresses MerTK/TIM-4 expression; reduced phagocytic capacity of gastric mucosal macrophages	CagA → SHP2 activation → impaired receptor tyrosine phosphorylation → attenuated downstream signaling	Gastric mucosal biopsy-based macrophage functional studies (71, 73).	Chronic atrophic gastritis; delayed mucosal repair; elevated risk of pre-neoplastic transformation
NAFLD/NASH	↓Kupffer cell MerTK; ↑circulating sMerTK (ADAM17 cleavage product); deficiency of RvD1 and other SPMs	Lipotoxicity → ER stress → ↑ADAM17 activity; ↓15-LOX-1 expression → reduced SPM biosynthesis	sMerTK levels positively correlate with NASH histological activity scores (90).; RvD1 enhances efferocytosis in NASH models (54)	Amplification of hepatocyte lipoapoptosis; accelerated progression of hepatic fibrosis
Pancreatitis (acute and chronic)	↓Pancreatic macrophage efferocytic capacity; NET-driven NLRP3 inflammasome activation	NETs activate trypsinogen; NLRP3 inflammasome activation; impaired clearance of necrotic acinar cells	Role of neutrophils/NETs in acute pancreatitis (85); macrophage polarization studies in chronic pancreatitis (87).	Expansion of acinar necrosis; pancreatic fibrotic remodeling; progression to chronic pancreatitis

ADAM17, a disintegrin and metalloproteinase 17; CD47, cluster of differentiation 47; ER, endoplasmic reticulum; IBD, inflammatory bowel disease; IL-10R, interleukin-10 receptor; LOX, lipoxygenase; MerTK, Mer tyrosine kinase; MFG-E8, milk fat globule-EGF factor 8; NAFLD, nonalcoholic fatty liver disease; NASH, nonalcoholic steatohepatitis; NET, neutrophil extracellular trap; NF-κB, nuclear factor kappa B; NLRP3, NOD-, LRR- and pyrin domain-containing protein 3; RvD1, resolvin D1; scRNA-seq, single-cell RNA sequencing; SHP2, Src homology region 2 domain-containing phosphatase 2; sMerTK, soluble MerTK (shed ectodomain); SPM, specialized pro-resolving mediator; STAT3, signal transducer and activator of transcription 3; TIM-4, T cell immunoglobulin and mucin domain-containing protein 4; UC, ulcerative colitis; CD, Crohn’s disease.

† Defective efferocytosis is not a uniform phenomenon across these four diseases but manifests through both shared and disease-specific mechanisms. The IL-10R→STAT3 signaling defect and ADAM17-mediated receptor shedding represent convergent pathological nodes across IBD and NASH (10, 34, 90), whereas *H. pylori* CagA-mediated receptor suppression (71, 73) and NET-driven NLRP3 activation (85) constitute disease-specific mechanisms in chronic gastritis and pancreatitis, respectively. In all four conditions, the shared downstream consequence of efferocytic failure—secondary necrosis of uncleared apoptotic neutrophils with subsequent DAMP release—establishes a self-reinforcing cycle of persistent inflammation that represents a common and therapeutically actionable endpoint (6, 9, 158).

## 4 Natural products modulating neutrophil efferocytosis

**Figure 4:**
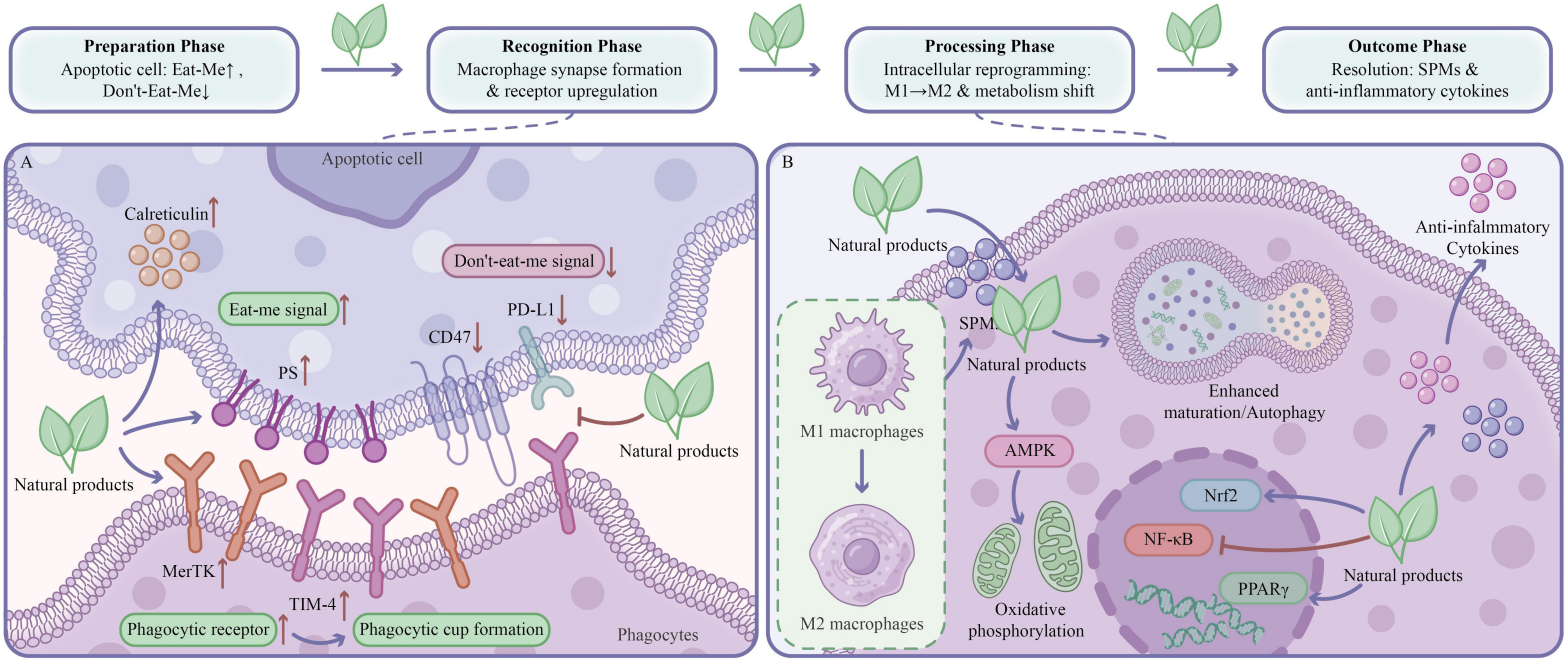
Natural product-mediated enhancement of efferocytosis and intracellular reprogramming of macrophages. Natural products restore efferocytic competence across four progressive phases (Preparation, Recognition, Processing, and Outcome). **(A)** Synapse formation and receptor modulation. On the apoptotic cell surface, natural products promote calreticulin surface translocation and augment PS externalization, amplifying “eat-me” signals. Simultaneously, they suppress “don’t-eat-me” signaling by downregulating CD47 and PD-L1, relieving SIRPα-mediated inhibition on the phagocyte. On the phagocyte membrane, natural products upregulate efferocytic receptors (MerTK and TIM-4) to enhance the efficiency of apoptotic cell recognition and internalization. **(B)** Intracellular reprogramming and inflammation resolution. Natural products converge on multiple intracellular nodes to shift macrophages from a pro-inflammatory M1 state toward a pro-resolving M2 phenotype. They activate AMPK to promote oxidative phosphorylation and restore metabolic support, while enhancing phagosome-lysosome fusion and autophagic flux for efficient cargo degradation. Within the nucleus, natural products activate Nrf2 and PPARγ to upregulate antioxidant defenses and efferocytic receptors, while concurrently inhibiting NF-κB to reduce pro-inflammatory cytokine transcription. The integrated outcome is enhanced secretion of anti-inflammatory cytokines and increased production of SPMs, driving the resolution of inflammation. PD-L1, programmed death-ligand 1; TIM-4, T-cell immunoglobulin and mucin domain containing 4; AMPK, AMP-activated protein kinase; Nrf2, nuclear factor erythroid 2-related factor 2; PPARγ, peroxisome proliferator-activated receptor gamma.

Natural products, with their structural diversity and multi-target properties, represent potential sources for developing novel efferocytosis modulators. Natural products can influence efferocytosis processes at multiple levels: regulating efferocytic receptor expression, affecting “eat-me” and “don’t-eat-me” signals, modulating macrophage polarization states, and improving the inflammatory microenvironment. This chapter elucidates natural product categories with substantial research support and their mechanisms of action. **Figure 4A** illustrates the biological processes in which these pathways are involved. The core efferocytosis mechanisms, key signaling pathways, levels of preclinical and clinical evidence, and primary limitations for each natural product class are summarized in **Table 3**, providing an integrated reference framework for the mechanistic discussions that follow.

**Table 3:** Natural products targeting neutrophil efferocytosis: mechanisms, signaling pathways, and evidence levels.

Compound class	Representative agents	Core efferocytosis mechanism	Key signaling pathways	Preclinical evidence	Clinical evidence	Primary limitations
Flavonoids	Quercetin, luteolin	↑MerTK/TIM-4 expression; ↑M2 macrophage polarization	PPARγ→LXRα; NF-κB↓	Murine colitis models; LPS-stimulated macrophage assays (100, 102, 103).	Limited; no efferocytosis-specific endpoints available (101).	Low oral bioavailability; extensive intestinal first-pass metabolism (101).
Terpenoids	Ginsenoside Rb1, glycyrrhizic acid, artemisinin	HMGB1 neutralization (glycyrrhizic acid); ↑macrophage phagocytic capacity; ↓DAMP signaling	NF-κB↓; MAPK↓; direct HMGB1 binding (107)	NASH and colitis rodent models (104–109)	Minimal; no efferocytosis-specific endpoints	Structural diversity limits mechanistic generalization; efferocytosis-specific data largely indirect; inter-species extrapolation caveats (157)
Polyphenols	Curcumin, resveratrol, EGCG	↓Oxidative PS modification; ↑M2 polarization; ↑efferocytic gene expression	Nrf2-HO-1; SIRT1; NF-κB↓	NASH and IBD murine models (110–112, 114, 115, 153) (155)	Metabolic endpoints supported by RCTs; efferocytosis-specific clinical endpoints lacking (113).	Poor oral absorption; rapid hepatic metabolism; inter-species extrapolation caveats (157)
Alkaloids	Berberine, matrine	AMPK activation → ↑OXPHOS → M2 polarization; ↑efferocytic function	AMPK-mTOR; NF-κB↓	NAFLD rodent models; *in vitro* macrophage studies (118).	Metabolic improvement RCTs for berberine (T2DM and NAFLD); efferocytosis-specific endpoints lacking (116, 117)	Narrow therapeutic window; CYP450-mediated drug interactions
Polysaccharides	APS, LBP, lentinan, GLP	Moderate macrophage activation via PRRs; ↑phagocytic capacity	TLR4-NF-κB (moderate activation); Syk-CARD9	Multiple inflammation models (119–121, 123).	Minimal overall; lentinan as adjuvant in gastric cancer chemotherapy (122); no efferocytosis-specific endpoints	Efferocytosis-specific data limited; substantial batch-to-batch structural variability
ω-3 PUFAs/SPMs	EPA, DHA, RvD1, LXA4	Direct SPM precursors; ↑MerTK/LXR expression; activation of ALX/FPR2 and GPR32 receptors	ALX/FPR2→cAMP→PKA; LXR→efferocytic gene transcription (52, 53, 132, 135, 137)	UC murine models (55, 56).; NASH murine models (54); RvD1-mediated PMN recruitment inhibition (154)	Cardiovascular RCTs for EPA (REDUCE-IT) (131); IBD secondary endpoint data (127, 128); efferocytosis-specific RCTs lacking	SPM chemical instability *in vivo*; route-of-administration and dose optimization unresolved; inter-species extrapolation caveats (157)

Efferocytosis-related evidence for most natural product classes derives predominantly from murine models. Marked genomic divergence between murine and human inflammatory responses (157) necessitates caution when extrapolating preclinical findings to clinical contexts. APS, Astragalus polysaccharides; CI-MPR, cation-independent mannose-6-phosphate receptor; DAMP, damage-associated molecular pattern; EGCG, epigallocatechin gallate; GLP, *Ganoderma lucidum* polysaccharides; IBD, inflammatory bowel disease; LBP, *Lycium barbarum* polysaccharides; NASH, nonalcoholic steatohepatitis; NET, neutrophil extracellular trap; OXPHOS, oxidative phosphorylation; PRR, pattern recognition receptor; PS, phosphatidylserine; RCT, randomized controlled trial; SPM, specialized pro-resolving mediator; UC, ulcerative colitis.

### 4.1 Flavonoids

As a class, flavonoids enhance efferocytosis through two mechanistically converging actions: (i) transcriptional upregulation of efferocytic receptors—most prominently MerTK and TIM-4—via activation of the nuclear receptor cascade PPARγ→LXRα→efferocytic gene cluster, which restores phagocytic machinery in macrophages exposed to pro-inflammatory microenvironments; and (ii) suppression of NF-κB-driven inflammatory gene transcription, which rebalances the macrophage phenotype away from the efferocytosis-hostile, receptor-shedding M1 state. Individual flavonoids differ primarily in receptor binding affinity, tissue distribution, and oral bioavailability, but their convergence on the PPARγ and NF-κB nodes constitutes the unifying mechanistic principle through which this class modulates efferocytosis. The following subsections describe representative members within this mechanistic framework.

Flavonoids are widely distributed polyphenolic secondary metabolites in plants, featuring a basic skeleton of two benzene rings (A and B rings) connected by a three-carbon chain. Based on the oxidation degree and cyclization pattern of the three-carbon chain, flavonoids are classified into subclasses including flavones, flavonols, flavanones, and isoflavones. These compounds possess antioxidant and anti-inflammatory activities, with some members potentially influencing efferocytosis through macrophage function modulation.

Quercetin (3, 3’, 4’, 5, 7-pentahydroxyflavone) is one of the most widely distributed flavonols in nature, abundantly present in onions, apples, tea, and berries. Quercetin exhibits multiple biological activities, including antioxidant, anti-inflammatory, and cell signaling modulation (100). The anti-inflammatory mechanisms of quercetin involve inhibiting NF-κB pathway activation, reducing transcription of pro-inflammatory cytokines. Quercetin may influence macrophage phenotype switching through nuclear receptor activation such as PPARγ, though its direct regulation of efferocytosis requires further investigation. To date, no clinical trial has evaluated quercetin’s effect on efferocytosis as a primary endpoint in gastrointestinal inflammatory disease; available evidence is restricted to *in vitro* and preclinical rodent studies, and the predominant glycoside forms encountered *in vivo* after oral administration may differ pharmacologically from the aglycone tested experimentally.

Quercetin bioavailability is significantly affected by its glycosylation degree. Free quercetin possesses better cell membrane permeability due to higher lipophilicity, though it naturally exists predominantly in glycoside forms (such as rutin and quercitrin) (101). Orally ingested quercetin glycosides are absorbed after hydrolysis to free quercetin in the small intestine and colon, or metabolized by gut microbiota into various phenolic acid metabolites. These metabolites may possess independent biological activities.

Luteolin, apigenin, and other flavonoids similarly possess anti-inflammatory activities (102). *In vitro* studies indicate that these compounds can attenuate oxidative stress in cell culture systems and may influence macrophage polarization states; however, direct evidence that luteolin or apigenin enhances efferocytosis in preclinical models or clinical settings remains limited, and clinical trials with efferocytosis-specific endpoints are lacking. Structural features of flavonoids (such as hydroxyl group positions on the B ring) correlate with their biological activities (103). Particularly, the 3’, 4’-catechol structure can exert powerful antioxidant effects through metal ion chelation and free radical scavenging.

### 4.2 Terpenoids

As a structurally diverse class, terpenoids enhance efferocytosis through two complementary mechanisms rooted in their ability to modulate the extracellular and intracellular inflammatory milieu: (i) neutralization of damage-associated molecular patterns (DAMPs)—most notably HMGB1—released by secondarily necrotic, uncleared apoptotic cells, thereby interrupting DAMP-driven activation of TLR4 and RAGE on phagocytes and breaking the self-reinforcing cycle of efferocytic failure and sterile inflammation; and (ii) suppression of NF-κB and MAPK signaling cascades within macrophages, which relieves transcriptional repression of efferocytic receptor genes (including *MERTK* and *TIMD4*) and facilitates polarization toward pro-resolving phenotypes competent for apoptotic cell uptake. Individual terpenoids—spanning triterpene saponins, diterpene lactones, and sesquiterpene lactones—differ markedly in structural complexity and primary molecular targets, yet their convergence on DAMP neutralization and NF-κB/MAPK suppression constitutes the unifying mechanistic principle through which this class supports efferocytosis. The following subsections describe representative members within this framework.

Terpenoids are natural products formed by isoprene unit polymerization, representing the most structurally diverse class of plant secondary metabolites. Based on isoprene unit numbers, terpenoids are classified as monoterpenes (C10), sesquiterpenes (C15), diterpenes (C20), triterpenes (C30), etc. Some terpenoids possess immunomodulatory activities, potentially influencing inflammatory responses and macrophage function through multiple mechanisms.

Triterpene saponins are amphipathic compounds formed by triterpenoid aglycones linked to sugar chains, possessing surface activity. Ginsenosides, the major active constituents of ginseng, are classified into protopanaxadiol-type (such as Rb1, Rb2, Rc, Rd) and protopanaxatriol-type (such as Rg1, Re, Rf) based on aglycone structure. Research in preclinical models indicates that ginsenosides can modulate macrophage function and influence inflammatory responses. Different ginsenoside types may act through distinct signaling pathways; however, whether this mechanistic selectivity translates into clinically meaningful differences in efferocytosis-related outcomes has not been established in controlled human trials (104). Different ginsenoside types may act through distinct signaling pathways, providing selectivity for clinical applications (105).

Astragaloside IV is the major triterpene saponin constituent of Astragalus membranaceus, possessing anti-inflammatory, antioxidant, and immunomodulatory properties (106). Astragaloside IV demonstrates protective effects in various preclinical inflammatory and tissue injury models. Its mechanism of action may involve regulation of multiple signaling pathways, including NF-κB, MAPK, and PI3K/Akt pathways; direct clinical evidence for efferocytosis-enhancing effects in gastrointestinal inflammatory diseases is currently absent. Its mechanism of action may involve regulation of multiple signaling pathways, including NF-κB, MAPK, and PI3K/Akt pathways.

Glycyrrhizic acid, the major triterpene saponin of licorice, is formed by glycyrrhetinic acid linked to two glucuronic acid molecules. Research confirms glycyrrhizic acid can directly bind high mobility group box 1 protein (HMGB1), blocking its interaction with pattern recognition receptors TLR4 and RAGE (107). HMGB1 is the major damage-associated molecular pattern (DAMP) released after cell necrosis, playing a key role in efferocytosis defect-associated inflammatory amplification. In biochemical and preclinical studies, glycyrrhizic acid directly binds HMGB1 and has been shown to attenuate HMGB1-driven inflammation in rodent models of secondary necrosis; whether this translates into improved efferocytosis in clinical gastrointestinal inflammatory disease remains to be demonstrated in dedicated trials. Additionally, glycyrrhizic acid and its metabolite glycyrrhetinic acid possess glucocorticoid-like effects, exerting anti-inflammatory effects through multiple mechanisms.

Andrographolide, the major diterpene lactone constituent of Andrographis paniculata, is traditionally used for treating infections and inflammation. Andrographolide possesses multiple biological activities, including anti-inflammatory, antioxidant, and potential anti-tumor effects (108). Its anti-inflammatory mechanisms involve inhibition of NF-κB and MAPK signaling pathways.

Artemisinin and its derivatives, extracted from Artemisia annua, are sesquiterpene lactone compounds renowned for their potent antimalarial activity. Beyond antimalarial effects, artemisinin compounds demonstrate immunomodulatory effects (109). In preclinical studies, artemisinin and its derivatives have been shown to influence immune cell function through multiple mechanisms, including cytokine production regulation and macrophage polarization; their direct effect on efferocytosis in gastrointestinal inflammatory disease contexts has not been evaluated in clinical trials. These mechanistic findings derive primarily from *in vitro* and preclinical animal studies; clinical evidence specifically addressing efferocytosis enhancement or resolution of gastrointestinal inflammation as primary endpoints is not yet available.

### 4.3 Polyphenols

Polyphenols as a class promote efferocytosis principally through two thematic mechanisms: (i) activation of the Nrf2-HO-1 antioxidant axis, which reduces oxidative modification of phosphatidylserine (PS) on apoptotic cell surfaces—thereby preserving PS recognition by MerTK and TIM-4—and simultaneously suppresses reactive oxygen species-driven shedding of efferocytic receptors by ADAM metalloproteases; and (ii) SIRT1-mediated deacetylation of NF-κB p65 and FOXO1, which shifts the macrophage transcriptional landscape toward anti-inflammatory, pro-resolving polarization with upregulated efferocytic receptor expression and enhanced SPM biosynthetic capacity. Curcumin, resveratrol, and EGCG each exemplify these mechanisms with distinct compound-specific pharmacological profiles as detailed below.

Polyphenolic compounds are plant secondary metabolites containing multiple phenolic hydroxyl groups, widely present in fruits, vegetables, tea, and spices. Polyphenolic compounds include simple phenolics, phenolic acids, lignans, stilbenes, and tannins among multiple subclasses. These compounds are renowned for their powerful antioxidant and anti-inflammatory activities.

Curcumin (1, 7-bis(4-hydroxy-3-methoxyphenyl)-1, 6-heptadiene-3, 5-dione), the major active constituent of Curcuma longa rhizomes, belongs to diarylheptanoid compounds. Curcumin exhibits extensive pharmacological activities, including anti-inflammatory, antioxidant, antimicrobial, and potential anti-tumor effects (110).

Curcumin’s anti-inflammatory mechanisms involve multiple signaling pathways. Curcumin can inhibit the NF-κB signaling pathway, the central regulatory pathway of inflammatory responses (111). Curcumin inhibits IκB kinase (IKK) activity, preventing IκB phosphorylation and degradation, thereby blocking NF-κB nuclear translocation and pro-inflammatory gene transcription. Additionally, curcumin can activate the Nrf2-ARE (antioxidant response element) pathway, upregulating antioxidant enzyme expression and enhancing cellular antioxidant capacity.

Curcumin may also influence inflammatory progression through macrophage polarization modulation. In preclinical cell culture systems and murine inflammation models, curcumin treatment promotes macrophage polarization toward M2-like phenotypes with upregulated efferocytic receptor expression. However, clinical evidence specifically demonstrating improved efferocytosis as an outcome in gastrointestinal diseases is currently lacking, and curcumin’s low oral bioavailability further limits the direct translation of these preclinical findings without delivery system optimization.

Curcumin’s clinical application is limited by its low bioavailability. Oral curcumin has low intestinal absorption and undergoes rapid hepatic metabolism to glucuronidated and sulfated conjugates. Strategies to enhance curcumin bioavailability include co-administration with piperine and development of novel delivery systems such as liposomes or nanoparticles.

Resveratrol (3, 5, 4’-trihydroxy-trans-stilbene), a natural stilbene compound, is widely present in grapes, blueberries, peanuts, and other plants. One of resveratrol’s primary mechanisms of action is activation of silent information regulator 1 (SIRT1), an NAD+-dependent histone deacetylase (112). SIRT1 regulates cellular metabolism, stress responses, and inflammation through deacetylation of multiple substrate proteins. Clinical trials of resveratrol have been conducted predominantly in metabolic disease contexts, and no randomized trial has assessed efferocytosis as a primary outcome in gastrointestinal inflammatory disease; furthermore, resveratrol’s rapid *in vivo* metabolism limits the direct translation of preclinical findings (113).

Green tea (Camellia sinensis) is rich in catechin polyphenols, primarily including epicatechin (EC), epigallocatechin (EGC), epicatechin gallate (ECG), and epigallocatechin gallate (EGCG). EGCG is the most abundant and active catechin in green tea (114). Tea polyphenols possess multiple biological activities, including antioxidant, anti-inflammatory, antimicrobial, and potential anti-tumor effects (115). EGCG’s antioxidant activity primarily derives from its multiple phenolic hydroxyl groups, which directly scavenge free radicals and chelate metal ions. Current evidence for EGCG’s effect on macrophage efferocytosis is confined to *in vitro* mechanistic studies; preclinical animal data and clinical trials specifically evaluating efferocytosis-related endpoints in gastrointestinal inflammatory diseases are lacking.

### 4.4 Alkaloids

As a class, alkaloids restore efferocytic competence in macrophages primarily through metabolic reprogramming converging on the AMPK–mTOR signaling axis: (i) activation of AMP-activated protein kinase (AMPK) promotes a shift from pro-inflammatory glycolytic metabolism toward oxidative phosphorylation (OXPHOS) and fatty acid oxidation (FAO), thereby supplying the sustained bioenergetic support required for phagosome formation, lysosomal acidification, and continual efferocytosis, while simultaneously upregulating transcription of efferocytic receptors through PGC-1α–driven mitochondrial biogenesis; and (ii) inhibition of NF-κB and MAPK pathways reduces the pro-inflammatory M1 transcriptional program that suppresses receptor expression and promotes ADAM metalloprotease-mediated efferocytic receptor shedding. These two axes—metabolic rebalancing and inflammatory signal attenuation—are mechanistically interdependent, as AMPK activation directly phosphorylates and inhibits IKKβ, linking energy sensing to NF-κB suppression in a unified regulatory circuit. Individual alkaloids such as berberine and matrine engage these nodes with distinct potencies and secondary targets, as described below.

Alkaloids are nitrogen-containing heterocyclic natural products, typically derived from amino acids, possessing basicity and diverse pharmacological activities. Alkaloids are widely distributed in plants, microorganisms, and animals, with complex and diverse structures including pyrrolidine, pyridine, isoquinoline, and indole types. Many alkaloids possess significant biological activities, occupying important positions in traditional medicine and modern drug development.

Berberine, an isoquinoline alkaloid, is the major active constituent of Coptis chinensis, Phellodendron amurense, and Berberis species. Berberine is bright yellow in color and traditionally used for treating gastrointestinal infections and diarrhea. One of berberine’s core mechanisms of action is activation of AMP-activated protein kinase (AMPK) (116). AMPK is the primary sensor of cellular energy status, activated during energy deficiency. In macrophages, AMPK activation promotes oxidative phosphorylation (OXPHOS) metabolism while inhibiting glycolysis, a metabolic shift favorable for M2 anti-inflammatory phenotype and efferocytic function maintenance.

Berberine has demonstrated metabolic efficacy—including glycemic control and reduction of hepatic steatosis—in randomized controlled trials involving type 2 diabetes and NAFLD patients (116, 117). However, these trials did not include efferocytosis-specific endpoints; berberine’s proposed efferocytosis-enhancing effects via AMPK activation in macrophages remain supported exclusively by preclinical studies and await dedicated clinical validation in gastrointestinal inflammatory disease contexts.

Matrine, a quinazoline alkaloid extracted from Sophora flavescens, is traditionally used for treating inflammatory diseases, liver diseases, and tumors. Matrine possesses anti-inflammatory, immunomodulatory, and anti-fibrotic effects (118). Its mechanism of action involves multiple signaling pathways, including NF-κB and MAPK pathway inhibition, Th1/Th2 balance regulation, and influence on apoptosis and autophagy. In preclinical inflammatory and fibrotic models, matrine demonstrates protective effects, potentially partially through macrophage function modulation. No clinical trial has evaluated matrine’s effect on efferocytosis as a defined endpoint in gastrointestinal inflammatory disease; clinical translation of these preclinical findings warrants dedicated investigation.

### 4.5 Polysaccharides

As a class, botanical and fungal polysaccharides modulate efferocytosis through pattern recognition receptor (PRR)-mediated phagocyte conditioning rather than direct intracellular signaling: (i) engagement of cell-surface PRRs—including Toll-like receptor 4 (TLR4), Dectin-1, and complement receptor 3 (CR3)—triggers calibrated macrophage activation that upregulates phagocytic receptor expression and lysosomal degradative capacity without driving the sustained pro-inflammatory M1 polarization that suppresses efferocytic function; and (ii) induction of trained immunity through epigenetic reprogramming at efferocytic gene loci, whereby prior polysaccharide exposure establishes a permissive chromatin state (characterized by increased H3K4me3 and H3K27ac marks at *MERTK*, *TIMD4*, and *MFGE8* promoters) that primes macrophages for enhanced apoptotic cell clearance upon subsequent inflammatory challenge. The net effect of this PRR-mediated conditioning is a sustained enhancement of basal efferocytic tone without the receptor-shedding and glycolytic reprogramming characteristic of classical inflammatory activation. Individual polysaccharides from *Astragalus membranaceus*, *Lycium barbarum*, *Lentinula edodes*, and *Ganoderma lucidum* engage overlapping yet distinct receptor repertoires and are described in turn below.

Polysaccharides are biological macromolecules formed by monosaccharides linked through glycosidic bonds, widely present in plants, fungi, bacteria, and animals. Plant and fungal polysaccharides have garnered attention for their immunomodulatory activities. These polysaccharides can activate innate immune cells through pattern recognition receptors (PRRs), influencing immune response direction and intensity.

Astragalus polysaccharides (APS), one of the major bioactive constituents of *Astragalus membranaceus*, are mainly composed of monosaccharides such as glucose, arabinose, and galactose. APS have been reported to exhibit immunomodulatory, antioxidant, and anti-fatigue activities (119). These immunomodulatory activities have been characterized primarily in preclinical studies; clinical evidence for APS-mediated enhancement of macrophage efferocytosis in gastrointestinal inflammatory disease is currently absent. Polysaccharides typically exert immunomodulatory effects through binding to cell surface pattern recognition receptors (such as Toll-like receptors, Dectin-1, complement receptor 3) (120).

Lycium barbarum polysaccharides (LBP), the major functional constituents of wolfberry, are heteropolysaccharides composed of various glycosyl units. LBP possess antioxidant, immunomodulatory, and neuroprotective effects (121). Lycium barbarum polysaccharides may moderately activate macrophages through receptors such as TLR4, enhancing their function without inducing excessive inflammatory responses. In preclinical studies, this ‘mild activation’ pattern has been associated with maintained macrophage phagocytic capacity without driving overt pro-inflammatory polarization; whether this translates into enhanced efferocytosis in human gastrointestinal inflammatory disease has not been clinically evaluated.

Lentinan, extracted from Lentinula edodes, is a β-1, 3-glucan possessing immunomodulatory and anti-tumor activities (122). Lentinan is recognized by macrophages and dendritic cells through Dectin-1 (a C-type lectin receptor) and complement receptor 3 (CR3), activating the Syk-CARD9 signaling pathway, inducing pro-inflammatory cytokine production and enhanced phagocytic function. These receptor-signaling interactions and phagocytic enhancement have been characterized in *in vitro* and preclinical settings; dedicated clinical evaluation of lentinan’s effect on efferocytosis in gastrointestinal inflammatory disease is lacking.

Ganoderma lucidum polysaccharides (GLP), one of the major bioactive constituents isolated from the fruiting bodies of Ganoderma lucidum, mainly consist of β-glucans and structurally diverse heteropolysaccharides (123). Ganoderma lucidum polysaccharides possess immunomodulatory, antioxidant, and hepatoprotective effects. In preclinical models, fungal polysaccharides have exhibited bidirectional immunomodulatory properties: enhancing immune function in immunosuppressed contexts and potentially modulating trained immunity in pro-inflammatory states. Clinical trials examining GLP’s effect on macrophage efferocytosis as a primary endpoint in gastrointestinal inflammatory disease are currently absent, and the trained immunity hypothesis specifically requires prospective clinical validation.

### 4.6 Omega-3 fatty acids and specialized pro-resolving mediators

Omega-3 polyunsaturated fatty acids (n-3 PUFAs) and their enzymatically derived specialized pro-resolving mediators (SPMs) occupy a unique mechanistic position among efferocytosis-enhancing natural agents: rather than modulating upstream inflammatory signaling, they function as direct biochemical substrates and receptor ligands within the resolution program itself. Their class-level contributions to efferocytosis operate through two synergistic mechanisms: (i) transcriptional upregulation of efferocytic receptors and SPM biosynthetic enzymes via n-3 PUFA-mediated activation of nuclear receptors PPARα, PPARγ, and LXR, which drive coordinated expression of MerTK, CD36, ABCA1, and 12/15-lipoxygenase, thereby simultaneously enhancing apoptotic cell recognition and increasing endogenous SPM biosynthetic capacity; and (ii) direct receptor-level amplification of efferocytosis by bioactive SPMs—resolvins, protectins, lipoxins, and maresins—acting through cognate GPCRs (ALX/FPR2, GPR32, ChemR23, LGR6) to activate Rac1-dependent phagocytic cup formation, promote phagosome–lysosome fusion, and stimulate continual efferocytosis through an autocrine feed-forward loop. This dual action—transcriptional priming and receptor-level activation—makes the n-3 PUFA/SPM axis the most proximally efferocytosis-coupled of all natural product classes reviewed here. The following subsections address n-3 PUFAs and individual SPM families in turn.

#### 4.6.1 Omega-3 polyunsaturated fatty acids

Omega-3 polyunsaturated fatty acids (n-3 PUFAs) are essential fatty acids that cannot be synthesized *de novo* by the human body and must be obtained from diet. Major n-3 PUFAs include α-linolenic acid (ALA, 18:3 n-3), eicosapentaenoic acid (EPA, 20:5 n-3), and docosahexaenoic acid (DHA, 22:6 n-3). ALA is primarily found in plant oils (such as flaxseed oil and walnut oil), while EPA and DHA are mainly sourced from marine fish and algae.

n-3 PUFAs are precursor substances for specialized pro-resolving mediators (SPMs) (8). At inflammatory sites, EPA and DHA are converted through enzymatic actions of lipoxygenases (particularly 5-LOX, 12-LOX, and 15-LOX) and cyclooxygenase-2 (COX-2, in the presence of aspirin) into bioactive lipid mediators (124). These SPMs include resolvins, protectins, and maresins, which play crucial roles in the resolution phase of inflammation (52).

n-3 PUFAs’ biological effects involve multiple mechanisms. First, n-3 PUFAs integrate into cell membrane phospholipids, affecting membrane fluidity, lipid raft structures, and membrane protein function (125). Increased n-3 PUFA content in cell membranes can alter signaling platform composition, influencing activation of multiple signaling pathways. Second, n-3 PUFAs can serve as ligands for nuclear receptors (such as PPARα, PPARγ, LXR, RXR), directly regulating gene transcription (126). Third, n-3 PUFAs reduce generation of pro-inflammatory eicosanoids through competitive inhibition of arachidonic acid (AA, 20:4 n-6) metabolism (127).

Epidemiological studies and clinical trials provide evidence for n-3 PUFAs’ health effects. High n-3 PUFA intake is associated with reduced risk of multiple chronic diseases, including cardiovascular diseases, certain cancers, inflammatory bowel diseases, and neurodegenerative diseases (128). Dietary n-3/n-6 PUFA ratios correlate negatively with inflammatory marker levels (129).

Western dietary patterns are characterized by imbalanced n-6/n-3 PUFA ratios (typically 15-20:1, while recommended ratio is 2-4:1). This imbalance may promote pro-inflammatory states, as n-6 PUFAs (particularly arachidonic acid) are precursors for pro-inflammatory eicosanoids.

Different n-3 PUFA types exhibit specific effects. EPA is primarily metabolized to resolvin E series (RvE1, RvE2, RvE3), while DHA is primarily metabolized to resolvin D series (RvD1-RvD6), protectin D1 (PD1/NPD1), and maresins (MaR1, MaR2) (130). High-dose EPA supplementation (>2 g/d) has demonstrated significant cardiovascular protection in specific clinical populations (REDUCE-IT trial). Direct clinical evidence for efferocytosis-specific benefits of omega-3 PUFA supplementation in gastrointestinal inflammatory diseases remains limited to exploratory or secondary endpoints; dedicated randomized trials with efferocytosis functional assessment as a primary outcome measure are warranted before clinical translation can be recommended (131). **Figure 4B** summarizes the mechanisms by which natural products promote macrophage polarization from the M1 to the M2 phenotype and enhance SPM generation via key pathways, including PPARγ, AMPK, and NF-κB.

#### 4.6.2 Specialized pro-resolving mediators

Specialized pro-resolving mediators (SPMs) are a class of bioactive lipid mediators generated through enzymatic conversion of n-3 and n-6 polyunsaturated fatty acids. Unlike traditional anti-inflammatory drugs that inhibit pro-inflammatory mediator generation, SPMs actively promote inflammation resolution and tissue repair processes (132). Inflammation resolution is not passive inflammatory decay but an actively regulated process controlled by SPMs, anti-inflammatory cytokines, and pro-resolution cellular programs.

Lipoxins: Lipoxin A4 (LXA4) and lipoxin B4 (LXB4) are the earliest discovered SPMs, generated from arachidonic acid through sequential action of 15-LOX and 5-LOX (133). Lipoxins act through the ALX/FPR2 receptor. Major biological effects of LXA4 include inhibiting neutrophil chemotaxis and transendothelial migration, promoting monocyte recruitment without activating their pro-inflammatory functions, enhancing macrophage phagocytosis (efferocytosis) of apoptotic neutrophils, and stimulating epithelial and endothelial cell repair. Exogenous LXA4 demonstrates inflammation resolution-promoting effects in multiple inflammatory models (134).

Resolvins: Resolvins are classified into E series (RvE1-3, derived from EPA) and D series (RvD1-6, derived from DHA). Resolvin D1 (RvD1), the most extensively studied resolvin, is produced through 15-LOX conversion of DHA to 17S-HpDHA, subsequently transformed by 5-LOX to RvD1 (135). RvD1 acts through two G protein-coupled receptors: ALX/FPR2 and GPR32. RvD1 can significantly enhance macrophage efferocytic capacity, promoting clearance of apoptotic neutrophils (136). In periodontitis, colitis, and acute lung injury models, RvD1 demonstrates significant pro-resolution and tissue protection effects (137).

Resolvin E1 (RvE1) is generated from EPA through sequential action of aspirin-acetylated COX-2 and 5-LOX (138). RvE1 primarily acts through ChemR23 receptor (also called ERV1 or CMKLR1) and BLT1 receptor. RvE1 can inhibit neutrophil migration through endothelial cells while promoting macrophage non-inflammatory phagocytic function (139). In asthma and acute lung injury models, RvE1 significantly reduces airway neutrophil infiltration and improves lung function (140).

Protectins/Neuroprotectins: Protectin D1 (PD1), termed neuroprotectin D1 (NPD1) in neural tissue, is generated from DHA through the 15-LOX pathway (141). PD1 is highly expressed and produced in the nervous system and ocular tissues (particularly retinal pigment epithelial cells). PD1 possesses powerful neuroprotective and anti-inflammatory effects, suppressing pro-inflammatory gene expression and promoting neuronal survival (142). In retinopathy and Alzheimer’s disease models, PD1 demonstrates protective effects (143).

Maresins: Maresins are so named because they are primarily generated by macrophages (macrophage mediators in resolving inflammation) (144). Maresin 1 (MaR1) is generated from DHA through the 12-LOX pathway. MaR1 acts through the LGR6 receptor. MaR1 can significantly enhance macrophage efferocytic capacity, promote macrophage conversion to pro-resolution phenotypes, and induce tissue regeneration-related gene expression. In peritonitis, obesity, and fatty liver models, MaR1 demonstrates inflammation resolution-promoting and metabolic improvement effects (145).

SPM Mechanisms of Action involve multiple cellular and molecular levels. First, SPMs inhibit neutrophil recruitment and migration to inflammatory sites (146). Second, SPMs enhance macrophage efferocytic capacity, promoting clearance of apoptotic neutrophils and preventing secondary necrosis and inflammatory amplification (59). Third, SPMs promote macrophage conversion to pro-resolution phenotypes (M2-like), which secrete anti-inflammatory factors and growth factors supporting tissue repair (54). Fourth, SPMs promote lymphocyte drainage, accelerating immune cell clearance from inflammatory sites (147).

Clinical Translation Challenges: Despite SPMs showing tremendous potential in multiple disease models, their clinical application still faces challenges. SPMs undergo rapid metabolism *in vivo* with short half-lives (typically minutes to hours) (148). Current clinical translation strategies include: developing metabolically stable SPM analogs; local administration; liposome or nanoparticle encapsulation; enhancing endogenous SPM biosynthesis capacity through precursor substance supplementation (n-3 PUFAs) (149).

Multiple preclinical studies support SPMs’ therapeutic potential. In limited clinical studies of periodontitis, local RvE1 application has been associated with periodontal tissue regeneration and reduced local inflammation; however, these findings are specific to the periodontal context and cannot be extrapolated to gastrointestinal inflammatory disease or used as evidence that RvE1 directly improves efferocytosis as a clinically measured outcome (150). In preclinical inflammatory bowel disease models, multiple SPMs (RvD1, RvD2, LXA4) have demonstrated mucosal healing-promoting and disease activity-improving effects; prospective clinical trials with efferocytosis or resolution biomarkers as primary endpoints in human gastrointestinal inflammatory disease have not yet been reported (56). In acute respiratory distress syndrome (ARDS), sepsis, and acute lung injury models, SPMs can reduce lung injury, lower inflammatory cytokine levels, and improve survival rates (151).

SPM Biosynthesis Dysregulation: Patients with chronic inflammatory diseases often exhibit imbalanced states of reduced SPM biosynthesis and relatively increased pro-inflammatory mediators (152). This imbalance may stem from multiple factors: precursor insufficiency (inadequate n-3 PUFA intake in modern Western diets with excess n-6 PUFAs); synthetic enzyme defects (inflammatory environments suppress 12/15-LOX expression or activity; certain populations have lipoxygenase gene polymorphisms); oxidative stress (excessive ROS can oxidize and inactivate SPMs) (153, 154). Correcting this imbalance may represent an important strategy for treating chronic inflammatory diseases.

### 4.7 Other active constituents

Caffeic acid phenethyl ester (CAPE), one of the major bioactive constituents of propolis, is a hydroxycinnamic acid derivative. CAPE has been reported to possess antioxidant, anti-inflammatory, and potential antitumor activities. Its anti-inflammatory effects are mainly attributed to inhibition of the NF-κB signaling pathway (155). In preclinical inflammatory and tissue injury models, CAPE demonstrates protective effects attributable to NF-κB inhibition; clinical evidence for CAPE-mediated efferocytosis enhancement in gastrointestinal inflammatory disease is currently lacking.

Allicin (diallyl thiosulfinate), the major organosulfur compound of garlic (Allium sativum), imparts the characteristic odor of freshly cut garlic. Evidence for allicin’s effects on macrophage efferocytosis derives from *in vitro* mechanistic studies; preclinical animal data and clinical trials evaluating allicin’s impact on efferocytosis-specific endpoints in gastrointestinal inflammatory disease are not currently available (156).

## 5 Challenges and future directions

Despite significant progress in research on natural products targeting efferocytosis for treating chronic inflammatory gastrointestinal diseases, translation from basic research to clinical applications still faces numerous challenges.

### 5.1 Limitations of current research

#### 5.1.1 Discrepancies between *in vitro* and *in vivo* models

Significant differences exist between *in vitro* cell models and *in vivo* pathological environments, which is a key issue constraining research translation (7). Traditional two-dimensional cell culture systems fail to simulate the complex tissue microenvironment *in vivo*, including cell-cell interactions, extracellular matrix components, and oxygen gradients. Natural products showing significant efferocytosis-enhancing activity *in vitro* may have diminished effects *in vivo* due to microenvironmental factors.

Animal models also have limitations. DSS-induced mouse colitis models primarily reflect acute epithelial injury rather than immune-mediated chronic inflammation characteristic of human IBD. While TNBS models more closely resemble Th1-mediated Crohn’s disease, they still cannot fully replicate the complexity of human disease. Different IBD models show significant differences in neutrophil dynamics, macrophage phenotypes, and efferocytosis efficiency.

Species differences are another important consideration. Gene expression profiles of inflammatory responses in mice differ significantly from humans (157). Although core molecular mechanisms of efferocytosis are relatively conserved among mammals, details of regulatory pathways and receptor expression patterns may differ.

#### 5.1.2 Bioavailability issues

Low bioavailability of natural products is a major obstacle to clinical translation. The absolute bioavailability of oral curcumin is extremely low, primarily due to poor water solubility leading to solubility-limited absorption, extensive hepatic metabolism due to first-pass effects, and rapid systemic clearance. Similar problems have been reported for quercetin, resveratrol, and many other natural products.

Even with high-dose administration, active compound concentrations reaching target tissues are often far below effective concentrations *in vitro* experiments. For colitis treatment, oral natural products must first withstand gastric acid and digestive enzymes, then be absorbed into the bloodstream, and finally reach inflamed intestinal tissue. Multiple losses during this process result in a huge gap between target tissue concentration and administered dose.

Metabolic conversion further complicates bioavailability issues. Most natural products are extensively metabolized *in vivo*, and generated metabolites may have activities different from parent compounds. Major metabolites of quercetin retain some activities of the parent compound but may have reduced activity in efferocytosis regulation.

#### 5.1.3 Target specificity

The multi-target nature of natural products is both an advantage and a challenge. While multi-target actions may produce synergistic effects, they also bring unclear mechanisms of action and potential off-target effects. The lack of clear molecular targets makes dose optimization and efficacy prediction difficult.

Certain flavonoid compounds, while promoting efferocytosis, may also increase infection risk through immune cell function inhibition. This “double-edged sword” effect suggests the need for more precise regulation of efferocytosis-related targets while avoiding adverse effects on host defense functions.

Additionally, different tissues and cell types may respond differently to the same natural product. Quercetin’s efferocytosis-regulating effects differ between intestinal macrophages and hepatic Kupffer cells, possibly due to differences in receptor expression patterns and signaling pathway activities across tissues.

### 5.2 Future research directions

#### 5.2.1 High-throughput screening strategies

Establishing efficient efferocytosis modulator screening platforms is key to accelerating drug discovery. Flow cytometry-based high-throughput efferocytosis detection methods can simultaneously evaluate hundreds of compounds’ efferocytosis-regulating activity in 96-well plate format. These methods use pH-sensitive fluorescent probe-labeled apoptotic cells to monitor phagosome acidification in real-time, distinguishing complete from incomplete efferocytosis.

AI-assisted virtual screening is another promising direction. Machine learning algorithms can learn structural features of known efferocytosis modulators and establish predictive models for screening natural product databases.

Phenotypic screening strategies may be more suitable for natural product research than target-directed screening. Since natural products often have multi-target actions, phenotypic screening can capture complex cellular effects without being limited to known targets.

#### 5.2.2 Nano-delivery system optimization

Regarding natural product bioavailability issues, developing nano-delivery systems is an important research direction. For intestinal inflammation, pH-responsive, enzyme-responsive, and inflammatory microenvironment-responsive nanocarriers show good targeting potential.

Smart responsive nanosystems represent a frontier direction in this field. ROS-responsive nanocarriers remain stable in normal tissues but release drugs at inflammatory sites (elevated ROS levels), achieving inflammation-specific delivery. Similar strategies can be applied to targeted delivery of other natural products.

Cell membrane-mimetic nanocarriers are an emerging delivery strategy. Macrophage membrane-coated nanoparticles can mimic natural cell biointerfaces, offering advantages of long circulation, low immunogenicity, and inflammation tropism. This biomimetic strategy is particularly suitable for delivering efferocytosis regulators targeting macrophage functions.

#### 5.2.3 Clinical translation research design

Transitioning from preclinical research to clinical trials requires careful design (57). Key considerations include reasonable patient selection criteria (such as stratification based on efferocytosis function), appropriate dosing timing (acute inflammation phase vs. remission phase), and reliable efficacy evaluation endpoints (not limited to symptom improvement but also including efferocytosis function indicators).

Biomarker-guided clinical trial designs may improve research efficiency. Incorporating peripheral blood efferocytosis function testing into enrollment criteria screens patient populations with efferocytosis defects for targeted treatment. This enrichment strategy may increase the likelihood of detecting therapeutic effects while identifying patient subgroups most likely to benefit.

### 5.3 Applications of emerging technologies

#### 5.3.1 Single-cell sequencing technology

Single-cell RNA sequencing (scRNA-seq) technology provides unprecedented resolution for characterizing efferocytosis-relevant cellular heterogeneity directly in disease tissues [60]. As a concrete application paradigm, scRNA-seq of lamina propria mononuclear cells from IBD patient biopsies can resolve intestinal macrophage populations based on the co-expression signature of efferocytosis-associated transcripts—MERTK, TIMD4, MFGE8, C1QA/B/C, and MARCO. This approach has already identified a disease-enriched macrophage subpopulation in active IBD lesions characterized by high IL-1β and TNF expression but near-complete transcriptional silencing of efferocytic genes—a profile that is entirely invisible in bulk RNA-sequencing (62). RNA velocity and pseudotime trajectory analysis can further map the differentiation path from recruited monocytes to tissue-resident macrophages, precisely identifying the differentiation stage at which efferocytic programming is derailed under inflammatory conditions. Applying this analytical framework to samples collected before and after natural product treatment (e.g., quercetin or berberine administration) would allow identification of the primary responder macrophage subpopulations and the molecular reprogramming events responsible for efferocytic restoration—providing cell-type-resolved mechanistic insights that bulk approaches cannot deliver.

#### 5.3.2 Spatial transcriptomics

Spatial transcriptomics technology can obtain gene expression data while preserving tissue spatial information. This is valuable for understanding spatial regulation of efferocytosis in tissue microenvironments (63). Spatial transcriptomics analysis of UC patient intestines found that efferocytosis defects mainly concentrate in actively inflamed areas and are closely related to local macrophage-neutrophil interactions.

Spatial omics technology can also evaluate tissue distribution and local effects of natural products. By combining drug imaging technology with spatial transcriptomics, associations between drug tissue distribution and local transcriptome changes can be established.

#### 5.3.3 Organoid models

Organoid technology provides a new platform for bridging *in vitro* research and *in vivo* applications. Intestinal organoids can reproduce three-dimensional structure and cellular composition of intestinal epithelium, representing an ideal model for evaluating effects of natural products on intestinal function. Intestinal organoid coculture systems containing immune cells can model intestinal inflammation and efferocytosis processes.

Organoid technology bridges the gap between reductionist *in vitro* assays and complex *in vivo* biology. A concrete three-step co-culture paradigm of particular value for efferocytosis research is as follows: (1) Establish patient-specific intestinal organoids from colonoscopic biopsies of IBD patients, retaining individual genetic backgrounds including disease-relevant IL-10R variants; (2) incorporate autologous macrophages derived from the same patient’s peripheral blood monocytes, differentiated under intestinal cytokine conditions (M-CSF + IL-10), into the three-dimensional Matrigel co-culture system in direct contact with the organoid epithelium; (3) quantify efferocytic function by adding pHrodo Red-labeled apoptotic neutrophils as phagocytic substrates, with uptake measured by live-cell confocal imaging and flow cytometry (pHrodo signal increases upon phagolysosomal acidification, providing a direct readout of completed efferocytosis). This system allows rigorous testing of whether candidate natural products—such as quercetin-loaded nanoparticles or berberine—can restore efferocytic capacity specifically in macrophages carrying patient-specific IL-10R signaling defects, while simultaneously monitoring the consequent changes in organoid epithelial barrier integrity (TEER measurement, tight junction immunofluorescence) and the local cytokine milieu (multiplex ELISA of conditioned medium). An analogous liver organoid co-culture platform incorporating Kupffer cell-like macrophages provides equivalent capability for NASH-focused efferocytosis studies.

## 6 Conclusions

Neutrophil efferocytosis defects have been established as a key pathological mechanism in chronic inflammatory gastrointestinal diseases. In diseases including inflammatory bowel disease, chronic gastritis, non-alcoholic fatty liver disease, and pancreatitis, clearance defects of apoptotic neutrophils lead to secondary necrosis, DAMP release, and sustained inflammation, forming a difficult-to-break pathological cycle. Restoration of efferocytosis function represents a novel therapeutic strategy for managing these diseases.

This review systematically outlined natural products regulating efferocytosis and their molecular mechanisms. Flavonoid compounds upregulate efferocytosis receptor expression through the PPARγ-LXR pathway; polyphenolic compounds promote macrophage polarization and efferocytosis function through the Nrf2-HO-1 pathway and SIRT1 activation; alkaloids regulate metabolic reprogramming and autophagy-dependent efferocytosis through the AMPK-mTOR pathway; terpenoid compounds and polysaccharides enhance phagocyte function through multiple mechanisms; omega-3 fatty acids directly participate in inflammation resolution programs as SPM precursors. The mechanisms of action of these natural products converge on several core signaling nodes—PPARγ, NF-κB, AMPK/mTOR, and Nrf2/HO-1 pathways.

From a translational medicine perspective, this review analyzed challenges and development directions in this field. Bioavailability optimization, targeted delivery strategy development, and clinical trial design are current priorities. Application of nano-delivery systems, prodrug strategies, and biomimetic carriers promises to overcome pharmacokinetic limitations of natural products. Combination applications with biologics and immunomodulators may produce synergistic effects. Personalized treatment strategies based on efferocytosis function assessment and molecular typing will provide patients with more precise treatments.

Applications of emerging technologies are deepening our understanding of efferocytosis mechanisms. Single-cell sequencing reveals heterogeneity of efferocytosis-related cells; spatial transcriptomics elucidates spatial regulation of efferocytosis in tissue microenvironments; organoid models provide *in vitro* evaluation platforms closer to human physiology. Integrated application of these technologies will accelerate discovery and translation of natural product efferocytosis regulators.

In conclusion, natural products targeting neutrophil efferocytosis provide new perspectives and strategies for treating chronic inflammatory gastrointestinal diseases. Although the path from laboratory to clinic still faces challenges, rapid development in this field and application of new technologies give us reason to expect that natural product treatments based on efferocytosis regulation will become effective treatment options for these refractory diseases in the near future.
